# Adenomyosis and Its Impact on Reproduction, Pregnancy, Childbirth, and the Newborn

**DOI:** 10.3390/ijms27219751

**Published:** 2026-10-30

**Authors:** Pietro Giulio Signorile

**Affiliations:** Italian Endometriosis Foundation, Via degli Olmetti 46, 00060 Formello, Rome, Italy; research@endometriosi.it

**Keywords:** adenomyosis, infertility, in vitro fertilization, miscarriage, pregnancy complications, preterm birth, placenta accreta spectrum, uterine rupture, neonatal outcomes, adenomyomectomy

## Abstract

This narrative review covers the diagnosis of adenomyosis; its impact on fertility, assisted reproduction, pregnancy, childbirth, and the newborn; and its uterus-sparing management. Diagnosis is delayed by about 11 years, and no classification is universally accepted. After in vitro fertilization, adenomyosis lowers clinical pregnancy and live birth rates and doubles miscarriage risk. Because oocyte number and morphology are preserved and miscarriage remains increased with donor oocytes and euploid embryos, the deficit appears mainly uterine; however, age and coexistent endometriosis contribute. In pregnancy, it is associated with preeclampsia, preterm birth, placental malposition and placenta accreta spectrum, cesarean delivery, and postpartum hemorrhage, and newborns are more often small for gestational age, of low birth weight, and extremely preterm. The evidence is observational and of low to very low certainty, and defective remodeling of the junctional zone spiral arteries remains a hypothetical unifying mechanism. In selected observational cohorts, pregnancy was reported in approximately half of infertile women after uterus-sparing surgery, which has not been compared with medical treatment or assisted reproduction in randomized trials and carries a risk of uterine rupture. Management rests on individualized counseling; proposed preventive measures, including low-dose aspirin and correction of vitamin D or folate deficiency, remain untested in adenomyosis.

## 1. Introduction

Adenomyosis, the presence of endometrial glands and stroma within the myometrium surrounded by hypertrophic smooth muscle, was historically a histological diagnosis made on hysterectomy specimens from multiparous women in their forties; imaging now identifies it in young women, often with endometriosis or fibroids, which coexist with it in 60–80% of cases [1].

**Search strategy and study selection.** PubMed/MEDLINE, Europe PMC, and publishers’ websites were searched up to September 2026, combining adenomyosis with infertility, in vitro fertilization, miscarriage, pregnancy outcome, preterm birth, preeclampsia, placenta, cesarean delivery, uterine rupture, neonatal outcome, anti-Müllerian hormone, oocyte, homocysteine, vitamin D, diagnosis, classification, conservative surgery, aspirin, and antenatal corticosteroids, and the reference lists of the retrieved reviews were screened. As a narrative review, this work followed no registered protocol and applied no formal risk-of-bias tool. Eligible sources were systematic reviews and meta-analyses, cohort and case–control studies, surgical series, clinical guidelines, and, for mechanisms, experimental studies; case reports were used only for uterine rupture, where no other source exists. When several syntheses addressed the same outcome, the most recent and largest and those grading the certainty of evidence were given priority, and earlier analyses were reported alongside them to show the direction and stability of the estimates; pooled estimates published between 2014 and 2026 were emphasized.

**Evidence appraisal.** Conflicting findings were weighed according to study design, sample size, adjustment for age, mode of conception, coexistent endometriosis or fibroids, and the imaging or histological criteria used; results are reported with the measure of association used by each study (odds ratio, OR; adjusted odds ratio, aOR; relative risk, RR; hazard ratio) and, where available, with absolute risks. Throughout the review, demonstrated associations are distinguished from proposed mechanisms and from clinical proposals specific to adenomyosis that have not been tested; this distinction is summarized in Section 8.

### 1.1. Epidemiology

Prevalence depends on population and diagnostic method [2]: diagnosed adenomyosis had an incidence of 28.9 per 10,000 woman-years and a prevalence of 0.8% in a United States cohort, with hysterectomy in 82%, figures that reflect clinical diagnosis rather than true prevalence [3]. Transvaginal ultrasound (TVUS) found it in 20.9% of general gynecology outpatients [4]. A meta-analysis estimated 10% in subfertile women [5], and magnetic resonance imaging (MRI) showed signs of adenomyosis in 79% of women with endometriosis, rising to 90% in women under 36 years with normal partner semen [6] (Figure 1).

A 2025 meta-analysis of 127 studies estimated a prevalence of about 1% (95% confidence interval [CI] 0–2%) in the general population, with much higher rates in selected women studied by histopathology (35.1%), MRI (35.0%), or ultrasound (30.7%), and about 42% in women with endometriosis and 31% in infertile women [7]. The association depends on phenotype: focal adenomyosis of the outer myometrium affected 50.2% of women with endometriosis versus 5.4% of controls and 66.3% of those with deep infiltrating endometriosis, whereas diffuse adenomyosis was not associated with any endometriosis phenotype [8].

### 1.2. Pathogenesis

Two hypotheses explain how endometrium comes to lie within the myometrium (Figure 2). The first involves invagination of the basal endometrium through a damaged endometrial–myometrial interface, which in the tissue injury and repair (TIAR) model is driven by chronic hyperperistalsis that causes microtrauma at the junctional zone, local estrogen production, and a self-perpetuating wound-healing response [9]. The second involves de novo metaplasia of Müllerian remnants or displaced stem cells. Next-generation sequencing shows that at least part of the disease derives clonally from the endometrial epithelium [10], with recurrent somatic KRAS mutations in 37.1% of cases [11].

The same junctional zone disruption is central to reproduction because this layer governs sperm transport, implantation, and the deep placentation on which fetal growth depends [12], a link that frames this review.

### 1.3. Classification

No classification is universally accepted (Table 1). Histological (Bird et al. [13]), MRI-based (Kishi et al.: intrinsic, extrinsic, intramural, and indeterminate types [14]; Chapron et al.: diffuse adenomyosis versus focal adenomyosis of the outer myometrium, FAOM [8]), clinical–histological (Grimbizis’s system, reviewed by Gordts et al.) [15], ultrasound- and MRI-based (Bazot and Daraï: internal, external, and adenomyoma) [16], and sonographic systems coexist. The latter includes the Morphological Uterus Sonographic Assessment (MUSA) reporting system, which describes location, type, cysts, layer, extent, and size without a grade [17], and the scores of Lazzeri et al. and Exacoustos et al., which define mild, moderate, and severe disease correlating with symptoms [18,19]. The #Enzian classification records adenomyosis (FA) with endometriosis [20], and microscopic adenomyosis identifies the earliest, histology-only form (Section 2) [21]. No multi-society consensus exists. The European Society of Human Reproduction and Embryology (ESHRE) declined to develop a consensus-based classification, holding that a useful system should derive from data [22], and deferred adenomyosis in its 2022 endometriosis guideline [23], whereas the 2026 single-society framework of the American & Global College of Endometriosis Specialists (AGCES) incorporates adenomyosis as an independent staging component (A1–A4) [24].

## 2. Diagnosis

Imaging has replaced histology for diagnosis in women of reproductive age, and consistent terminology is essential for comparing reproductive outcomes. The MUSA group standardized the sonographic description of myometrial lesions [25], and a modified Delphi revision distinguished three direct features, which reflect ectopic endometrium, from six indirect features, which reflect the myometrial response [26] (Figure 3).

MRI is valuable when TVUS is inconclusive or when fibroids distort the uterus, with a junctional zone of 12 mm or more and high-signal intramyometrial foci as the usual criteria [1]; an integrated approach combining symptoms (dysmenorrhea, heavy menstrual bleeding, subfertility) with TVUS first and MRI second is recommended (Figure 4).

Phenotype matters: lesions may be diffuse or focal (adenomyoma), internal (arising from the junctional zone) or external (arising from the serosa, often with deep endometriosis), and of variable extent, distinctions that are not yet applied uniformly in reproductive studies and that are a major source of heterogeneity in pooled estimates. Coexistent endometriosis, present in most women with adenomyosis [1], is a further confounder that few studies adjust for. Differences in imaging criteria (a junctional zone of 12 mm or more, a junctional zone difference of 5 mm or more, or the direct and indirect MUSA features), in lesion location and in disease extent therefore affect every association reported in Section 3, Section 4, Section 5 and Section 6, and the two main phenotypes do not behave alike. Focal lesions are more amenable to complete excision and carry better reproductive results after surgery (Section 7.1). However, focal adenomyosis hampered implantation and live birth after euploid embryo transfer more than diffuse disease (Section 3.7), and the preterm birth risk rose with uterine volume mainly in focal disease (Section 4.3). In contrast, diffuse disease has been linked to more preeclampsia and preterm premature rupture of membranes (PPROM; Section 4.4) and to lower pregnancy rates after surgery (Section 7.1). Where the primary studies allow, results are reported by phenotype.

Microscopic adenomyosis, proposed as the earliest form, consists of small, often multifocal glands within the myometrium, typically without stroma, that are missed at imaging and on routine histology; cytokeratin immunostaining identifies the glands, and the absence of CD10 staining confirms the lack of stroma, suggesting that the stromal component develops later. Recognizing it may explain symptomatic women with negative investigations and treatment failures, and its inclusion in classifications, with immunohistochemistry when suspicion is high, has been advocated [21]; because it escapes imaging, reproductive studies may misclassify affected women as controls, diluting the associations in Section 3, Section 4, Section 5 and Section 6.

**Diagnostic accuracy of ultrasound and MRI.** Against histology, TVUS has a pooled sensitivity of 72% and a specificity of 81%, with respective values of 77% and 89% for MRI, in an indirect comparison [27]. However, in a head-to-head meta-analysis of women who underwent both, performance was similar (TVUS 75% and 81%, MRI 69% and 80%) [28]. TVUS is cheaper, more widely available and the usual first-line test; the accuracy of both depends on operator and criteria and is lower for focal or early disease and in the presence of fibroids.

**Diagnostic delay.** Because symptoms are nonspecific and imaging criteria are recent, adenomyosis is recognized late. In the French ComPaRe-Endometriosis e-cohort (6949 women), the average delay was 11 years versus 10 for endometriosis. It was longer with comorbidities, severe dysmenorrhea, unemployment, and multiple consultations and shorter with a comfortable financial situation and an older age at menarche and at symptom onset [29]. This interval often covers the years of attempted conception, so early TVUS in women with dysmenorrhea, heavy menstrual bleeding, or infertility could shorten it.

## 3. Adenomyosis and Reproduction

### 3.1. Adenomyosis and Fertility

Adenomyosis is associated with roughly 40% lower odds of live birth after in vitro fertilization (IVF) and with twice the risk of miscarriage; the evidence comes almost entirely from assisted reproductive technology (ART) cohorts because natural fecundity is difficult to measure in unselected populations. MRI has shown a strong association with endometriosis in infertile women, with junctional zone thickening increasing with endometriosis stage and age [6], and the 10% prevalence among subfertile women makes adenomyosis relevant to every fertility service [5]. Data on spontaneous conception are indirect. No prospective study has measured fecundability in women with untreated adenomyosis. In a nationwide survey of infertile women, 41–43% conceived without pretreatment or surgery, a proportion similar to that after medical pretreatment or adenomyomectomy (Section 7.1) [30]. After uterus-sparing surgery, 30–41% of women attempting natural conception conceived within 3 years [31,32]. The population-based cohorts of Section 4 and Section 5 include pregnancies conceived both spontaneously and by ART, although few report outcomes separately by mode of conception [33,34].

### 3.2. Outcomes of Assisted Reproduction

The first meta-analysis found clinical pregnancy in 40.5% of women with adenomyosis versus 49.8% of those without (RR 0.72) and miscarriage in 31.9% versus 14.1% (RR 2.12) [35]. A subsequent one confirmed lower live birth (OR 0.59) and higher miscarriage (OR 2.20) [36]. Two later syntheses agreed on clinical pregnancy and miscarriage [37,38], with similar live birth estimates that were significant in one [38] but not in the other [37] (Figure 5). A broader analysis of adenomyosis and endometriosis found a threefold increase in miscarriage (OR 3.40) [39].

The consistent direction across syntheses that share many primary studies is reassuring. However, the primary studies are retrospective, diagnostic criteria vary, and adjustment for age, embryo quality, and endometriosis is inconsistent. Where these factors were addressed, the association persisted. Live birth remained lower in pairs matched for age and ovarian response [40] and after adjustment for age in first frozen blastocyst transfers (aOR 0.46) [41], and embryo quality did not differ in the two largest cohorts (Section 3.7) [40,41]. Two caveats remain. Endometriosis, which coexists in most cases, was not separated from adenomyosis in several cohorts [42], so part of the effect attributed to adenomyosis may reflect endometriosis. In addition, the treatment protocol matters since the benefit of gonadotropin-releasing hormone agonist (GnRHa) pretreatment was confined to fresh transfers (Section 3.3) [43], whereas the excess miscarriage was still seen after single euploid frozen transfer [44,45]. Diffuse disease and a larger uterine volume have been linked to a worse prognosis than focal lesions, but data by phenotype are sparse and conflicting (Section 3.7).

### 3.3. Medical Pretreatment Before ART

Prolonged pituitary downregulation with GnRHa aims to shrink lesions and reduce inflammation before transfer. An early retrospective study suggested a benefit before frozen embryo transfer (FET) [46]. However, pooled data are less encouraging, and the distinction between clinical pregnancy and live birth, the outcome that matters to patients, is decisive. In eight retrospective studies, pretreatment increased clinical pregnancy (OR 1.42) and implantation but not live birth [47]. Medical pretreatment improved clinical pregnancy after fresh but not after frozen transfer, with no effect on live birth or miscarriage [43]. A 2025 meta-analysis of 1914 FET cycles found no benefit for any outcome [48], as had an earlier subgroup analysis [38] (Figure 6). A higher clinical pregnancy rate that does not translate into more live births implies that losses after implantation are not prevented. Whether effectiveness differs by phenotype or severity is unknown because no study has stratified by lesion type, extent, or uterine volume, and the apparent benefit in fresh but not in frozen cycles [43] may reflect differences in endometrial preparation rather than an effect on the disease. Pending randomized trials, pretreatment should be individualized rather than routine (Section 7.2).

### 3.4. Mechanisms of Impaired Implantation

Reduced implantation may reflect several mechanisms (Figure 7): disruption of the junctional zone and hyperperistalsis, which impair sperm transport and embryo positioning [6,9]; reduced endometrial adhesion and receptivity molecules (Section 3.5); local inflammation and oxidative stress; and abnormal decidualization and vascularization. The junctional zone links these mechanisms to later obstetric disease because the same layer must be remodeled for normal placentation [12].

### 3.5. Endometrial Adhesion and Receptivity Molecules

The eutopic endometrium in adenomyosis expresses lower levels of the molecules that allow the blastocyst to appose and attach during the window of implantation. The trophoblast first rolls on the epithelium through L-selectin and its ligand and then attaches through integrin αvβ3 bridged by osteopontin, steps orchestrated by leukemia inhibitory factor (LIF) and the transcription factor HOXA10, each of which is altered in adenomyosis (Table 2).

LIF was lower in the endometrium and uterine flushing fluid of women with adenomyosis sampled 7–9 days after ovulation [53]. Reduced LIF receptor expression disrupts signal transducer and activator of transcription 3 (STAT3) signaling [54]. A 2024 immunohistochemical study confirmed lower stromal osteopontin, LIF, HOXA10, and progesterone receptor levels, with fewer pinopodes but no difference in integrin β3 [51]. These small tissue studies offer a molecular explanation for the lower implantation and live birth rates, although whether restoring these molecules improves implantation is unknown.

### 3.6. Ovarian Reserve and Anti-Müllerian Hormone

Adenomyosis may also be associated with reduced ovarian reserve. In 150 women assessed with three-dimensional TVUS and serum anti-Müllerian hormone (AMH), those with adenomyosis had lower AMH, a lower antral follicle count (AFC), and a smaller ovarian volume than controls, a reduction less marked than that seen with endometriomas. AMH correlated strongly with AFC and ovarian volume [56].

Age and endometriosis are the main confounders. Among women undergoing cystectomy for endometrioma, coexisting adenomyosis was associated with lower preoperative AMH (2.45 versus 3.19 ng/mL) and a greater postoperative decline, but not after adjustment for age [57]. A review of studies published between 2017 and 2022 concluded that the relationship remains unclear, although AMH tends to be lower and is not modified by adenomyomectomy, and that the AMH type II receptor is strongly expressed in adenomyotic tissue [58].

Experimental data suggest that the ovary itself can be affected, although not in adenomyosis directly. In a murine model in which perinatal exposure to bisphenol A, an endocrine disruptor, induces an endometriosis-like phenotype in female offspring, the ovaries at three months contained fewer primordial and developing follicles and more atretic follicles than those of unexposed controls of the same age, most markedly in mice with the phenotype [59]. Thus, age cannot explain the difference. This is a model of endocrine disruption and endometriosis-like disease, not of adenomyosis, and it has no direct counterpart in human data. It is cited here only as a hypothesis-generating observation that the lesions and a reduced follicle pool might share a developmental origin, which would need confirmation in women with adenomyosis before any clinical inference is drawn.

AMH also has prognostic value since younger age and higher AMH predicted pregnancy after adenomyomectomy [60]. Because women with adenomyosis are often older at diagnosis and frequently have endometriosis, ovarian reserve should be assessed with AMH and AFC at the first fertility consultation, as in any infertile woman, and a low value should prompt timely ART rather than prolonged expectant management or repeated surgery. Prospective studies adjusting for age and endometriosis are needed.

**Extra-ovarian production of AMH: an exploratory hypothesis.** AMH is not produced by the ovary alone. Deep endometriosis expresses AMH and its type II receptor (AMHRII), and recombinant AMH induces cell cycle arrest and apoptosis in endometriosis cells [61]. AMH and AMHRII are increased in the eutopic endometrium and lesions of women with endometriosis, although serum AMH did not differ from that of controls [62]. AMHRII is strongly expressed in adenomyotic tissue [63]. It has been hypothesized that serum AMH in extensive endometriosis, and possibly adenomyosis, might include a contribution from the lesions; thus, the true ovarian reserve would be lower than the serum value suggests. This hypothesis is speculative. Tissue expression of AMH or its receptor does not demonstrate secretion into the circulation. No study has measured such a contribution. The similar serum AMH level despite increased tissue expression [62] and the absence of change after adenomyomectomy [58] argue against a clinically relevant effect, although these studies were small. Measuring AMH in lesion tissue, uterine venous blood, and serum before and after complete excision of extra-ovarian disease with the ovaries preserved would test it; until then, it should not alter the clinical interpretation of AMH, which is used together with AFC, as in other infertile women.

### 3.7. Oocyte Quantity and Quality

Whether adenomyosis affects the oocyte or only the uterus matters for counseling and treatment, and most evidence points to the uterus. In 1146 matched pairs undergoing IVF, the numbers of oocytes, zygotes, and embryos did not differ, yet implantation and live birth rates were lower with adenomyosis [40]. The largest morphological analysis (205,978 oocytes) found no effect on oocyte morphology but more pregnancy losses and fewer live births, with a lower oocyte yield associated with endometriosis rather than adenomyosis [64]. Women with endometriosis and/or adenomyosis had the same number of mature oocytes but a cumulative live birth rate about 15% lower over three cycles [42]. In first frozen blastocyst transfers, embryo quality was similar, whereas clinical pregnancy (aOR 0.62) and live birth (aOR 0.46) were reduced [41].

Oocyte donation provides a natural experiment with oocytes from young donors. Adenomyosis did not affect implantation but doubled miscarriage in recipients [65]. In a prospective cohort of 228 recipients, implantation and live birth did not differ significantly, but miscarriage nearly doubled (35.4% versus 18.1%), most markedly when the junctional zone was involved [66]. With MUSA criteria, adenomyosis did not reduce live birth after donor oocyte transfer, although larger focal lesions did [67]. Even after single euploid blastocyst transfer, which removes aneuploidy as a factor, adenomyosis reduced implantation and live birth (24.6% versus 46.9%), particularly in its focal form (15% versus 28.3% for diffuse disease) [44].

Adenomyosis therefore does not appear to substantially reduce the number or morphological quality of oocytes and embryos, and the lower success of ART seems largely uterine in origin, mediated by impaired implantation and, above all, early pregnancy loss. The donor oocyte studies consistently show an increased miscarriage risk, whereas they do not consistently show lower implantation or live birth rates, which have instead been demonstrated after euploid embryo transfer. This conclusion should be drawn with caution. The studies are observational. Women with adenomyosis are older and more often have endometriosis and other infertility factors. Adjustment for these variables was incomplete, and the lower oocyte yield of some cohorts may reflect age, reduced ovarian reserve (Section 3.6), or endometriosis rather than adenomyosis itself. With that caveat, the evidence supports uterus-directed strategies, such as individualized pretreatment (Section 3.3) and frozen single embryo transfer, rather than efforts to increase oocyte yield, and indicates that oocyte donation alone does not overcome adenomyosis-related pregnancy loss.

## 4. Adenomyosis and Pregnancy

### 4.1. Pathophysiology

Defective deep remodeling of the myometrial spiral arteries in a disrupted junctional zone is the leading unifying hypothesis [12,68]. This is supported by histological and observational data rather than by direct demonstration in pregnancies with adenomyosis. Incomplete conversion of these arteries would cause shallow placentation and hypoperfusion, the substrate of preeclampsia, fetal growth restriction (FGR), and many spontaneous preterm births. Increased contractility and local inflammation may favor PPROM and preterm labor, and an enlarged, stiff, poorly contracting uterus may explain malpresentation, cesarean delivery, and postpartum hemorrhage (Figure 8). These are proposed causal pathways; the associations themselves are presented in Section 4.2, Section 4.3, Section 4.4 and Section 4.5.

**Homocysteine and folate metabolism.** Hyperhomocysteinemia has been proposed as an additional link, but the evidence is indirect and does not establish a causal role in adenomyosis. In a single small study, women with adenomyosis had higher serum homocysteine; lower 5-methyltetrahydrofolate (5-MTHF), the active form of folate; and a higher frequency of the heterozygous methylenetetrahydrofolate reductase (MTHFR) C677T genotype than controls, with widely dispersed values and no pregnancy outcome data [69]. In the general obstetric population, elevated maternal homocysteine is associated with the same complications that are increased in adenomyosis (preeclampsia, preterm birth, fetal loss, small for gestational age [SGA], and low birth weight), with low to very low certainty [70]. Homocysteine is toxic to the endothelium, induces trophoblast apoptosis and necrosis, reduces chorionic gonadotropin secretion in vitro [71], and may compete with other amino acids for placental transport [72]. Clinically, high maternal homocysteine has been associated with SGA [73]; with placenta-mediated complications and SGA but not with abruption [74]; and, in the Generation R cohort, with lower placental weight and birth weight and with SGA. Low folate had similar effects, and the combination carried the highest risk of SGA and preeclampsia [75]. No study has examined whether hyperhomocysteinemia contributes to obstetric risk in women with adenomyosis, and the two lines of evidence are joined here only by analogy. Periconceptional folic acid (400 µg daily) is recommended for all women who could become pregnant, regardless of MTHFR genotype, because the common MTHFR variants do not prevent the processing of folic acid and are not a reason to avoid it [76]. Whether measuring homocysteine or folate status in women with adenomyosis adds anything to this standard care is unknown. It is listed among the research questions in Section 8 and is not proposed as routine practice.

### 4.2. Miscarriage and Recurrent Pregnancy Loss

Miscarriage is the earliest and most consistent adverse outcome. After ART, meta-analyses report an approximate doubling of risk (RR 2.12, OR 2.20) [35,36] and an OR of 2.81, with no data for spontaneous conception [77]. The ADELIN analysis confirmed a smaller increase in IVF pregnancies (RR 1.61), whereas its overall estimate, which included unselected conceptions, was not significant [78]. Second-trimester miscarriage is a characteristic complication [79]. The excess persists with euploid embryos. In a retrospective series, miscarriage occurred in 53% of women with adenomyosis versus 19.7% of those without (aOR 3.7) and fell from 82.4% to 35.7% with ultra-long GnRHa downregulation [45]. The risk is also doubled in donor oocyte recipients [65,66].

Adenomyosis is more frequent in women with recurrent pregnancy loss (RPL): 38.2% versus 22.3% among infertile women examined with three-dimensional ultrasound [80] and 19.7% of women with recurrent miscarriage versus 6.1% of controls, including 13.6% whose losses were otherwise unexplained [81]. Location matters. In RPL with adenomyosis, lesions confined to the inner myometrium were more frequent than in women with adenomyosis without RPL (27.5% versus 10%), in whom outer-myometrium lesions predominated (65% versus 40%), and women with both conditions had more losses than those with RPL alone [82]. Coexistent endometriosis adds to the risk. Previous early miscarriage was reported in 28.5% of pregnancies versus 11.3% in controls, with the highest rate in deep endometriosis [83].

These data again point to the junctional zone, where defective spiral artery remodeling and abnormal myometrial function may compromise placentation from its earliest stages [12,68]. Women with recurrent miscarriage may reasonably be examined for adenomyosis by TVUS or MRI, with attention to the inner myometrium, and women with adenomyosis should be informed of the higher risk. Whether pretreatment reduces miscarriage is unproven (one retrospective series suggested a benefit, but pooled data do not; Section 3.3) and requires randomized trials.

### 4.3. Pooled Evidence

Meta-analyses have consolidated the cohort signals (Figure 9). An early synthesis found increased preeclampsia and preterm birth [84]. A larger review confirmed higher odds of preterm birth (OR 2.65), preeclampsia (OR 4.32), cesarean delivery, malpresentation, and postpartum hemorrhage [37]. The ADELIN analysis (39 studies, over 5.5 million pregnancies) found increased risks of preeclampsia (RR 2.24), placental malposition (RR 3.26), PPROM (RR 2.87), postpartum hemorrhage (RR 1.73), and, in IVF pregnancies, miscarriage (RR 1.61), whereas FGR, neonatal intensive care admission, and uterine rupture did not differ, with low to very low certainty throughout [78]. A 2026 meta-analysis of 25 cohorts found smaller but significant associations with preterm birth, placenta previa, preeclampsia, and postpartum hemorrhage [85].

Effect sizes shrink as studies become larger and more population based, suggesting that early estimates were inflated by the selection of severe, symptomatic, or ART-conceived cases, but the direction is consistent. The earliest cohorts from Asia reported independent associations with preterm delivery (aOR 1.96), spontaneous preterm delivery (aOR 1.84), and PPROM (aOR 1.98), with asymmetrical published confidence intervals [86]; markedly higher odds of preterm birth (OR 5.0), cesarean delivery (OR 4.5), malpresentation (OR 4.2), and postpartum hemorrhage (OR 6.5) in women with uterine enlargement [87]; and more second-trimester miscarriage (12.2% versus 1.2%), preeclampsia (18.3% versus 1.2%), and placental malposition (14.2% versus 3.2%), with very wide confidence intervals [79]. Extent modifies risk. Among 586 women with adenomyosis diagnosed before pregnancy, 15.2% delivered preterm, and each 10 cm^3^ of pre-pregnancy uterine volume raised the odds of preterm birth by 37% (aOR 1.37), with an inflection at about 89 cm^3^, in focal (aOR 1.43) but not in diffuse disease (aOR 1.10) [88].

### 4.4. Placental Pathology

Adenomyosis is associated with disorders of placental site, invasion, separation, and function (Table 3), of which abnormal location is the most consistent. In a nationwide Korean cohort, adenomyosis diagnosed before or during pregnancy raised the risk of placenta previa (aOR 1.50 and 1.70) and abruption (aOR 1.51 and 1.60) [89]. In a propensity-matched US analysis, it was associated with previa (aOR 5.08), placenta accreta spectrum (PAS; aOR 3.07), and abruption (aOR 3.21) [90]. After IVF, previa was almost three times more frequent (4.0% versus 1.4%) [40].

PAS occurred in 1.4% of women with adenomyosis versus 0.5% of those without in a Japanese nationwide study (OR 1.95), with 26.5% of the association mediated by ART [91]. The risk is highest after uterine surgery, with PAS complicating 9 of 18 pregnancies after adenomyomectomy versus none of 105 in women with untreated adenomyosis [92].

Lesion type and location matter. Diffuse disease carried more preeclampsia (25.0% versus 7.7%) and PPROM (19.4% versus 1.9%) than focal disease, and external adenomyosis was associated with placental malposition in 20% of cases [93]. Placental histopathology in adenomyosis has not been described systematically; among pregnancy-related hysterectomies, adenomyosis was found in 13.8% of uteri and was associated with more placenta previa and chorioamnionitis [94].

**Table 3:** Placental pathologies associated with adenomyosis.

Placental Pathology	Main Findings in Adenomyosis	Reference
Placenta previa or malposition	RR 3.26 (meta-analysis); RR 2.15 (meta-analysis); aOR 1.50–1.70 (population cohort); aOR 5.08 (matched cohort); 20% with external adenomyosis	[78,85,89,90,93]
Placenta accreta spectrum	1.4% versus 0.5%, OR 1.95; 2.0% versus 0.5%, OR 2.26; aOR 3.07; 50% after adenomyomectomy	[33,90,91,92]
Placental abruption	aOR 1.51–1.60; aOR 3.21	[89,90]
Retained placenta	aOR 1.29	[34]
Placental dysfunction (preeclampsia, SGA)	See Section 4.3, Section 4.5 and Section 6.1	[37,78]
Histopathology	No dedicated studies; more chorioamnionitis in pregnancy-related hysterectomies with adenomyosis	[94]

aOR, adjusted odds ratio; OR, odds ratio; RR, relative risk; SGA, small for gestational age.

### 4.5. Fetal Growth and Low Birth Weight

Impaired fetal growth is the most consistent fetal consequence. Beyond the meta-analyses in Figure 10 (Section 6.1), large cohorts confirm it: SGA in the Dutch population study (aOR 1.15) [34], FGR in the Japanese registry (5.3% versus 3.8%) [33], and SGA in a Korean cohort whether adenomyosis was diagnosed before (aOR 1.22) or during pregnancy (aOR 1.29) [89]. With uterine enlargement, the odds of SGA were more than fourfold higher [87].

Low birth weight has two sources that often coexist: preterm birth, which shortens the time available for growth, and placental insufficiency, linked to defective remodeling of the junctional zone spiral arteries [68]. Not every study confirms an excess of FGR [78], possibly because definitions and the timing of assessment differ.

Such pregnancies need closer monitoring. Serial third-trimester growth scans are reasonable when adenomyosis is known, especially in diffuse disease or with an enlarged uterus.

**Low-dose aspirin: established indications and an untested proposal.** Because the excess of FGR and preeclampsia appears to be mediated by defective placentation, prevention with low-dose aspirin, which is effective in other placental disorders, has been suggested for adenomyosis. The established indications must be kept distinct from this proposal. Aspirin started at or before 16 weeks reduces FGR (RR 0.56) and preeclampsia (RR 0.57) in women at high risk, with a dose–response effect, whereas aspirin started later does not affect growth [95]. In the Aspirin for Evidence-Based Preeclampsia Prevention (ASPRE) trial, 150 mg at night from 11–14 weeks until 36 weeks reduced preterm preeclampsia from 4.3% to 1.6% [96]. Guidelines recommend it for women with one high-risk or several moderate-risk factors for preeclampsia, for a longer duration than in ASPRE. The American College of Obstetricians and Gynecologists (ACOG) recommends 81 mg daily, started between 12 and 28 weeks of gestation (optimally before 16 weeks) and continued until delivery [97], and the UK National Institute for Health and Care Excellence (NICE) recommends 75–150 mg daily from 12 weeks until birth [98]. Adenomyosis is not a listed risk factor in any guideline. No trial has tested aspirin in this population, so its use cannot be presented as routine care for adenomyosis alone. It is proposed here, by extrapolation, as an individualized option after assessment of the overall risk profile, when adenomyosis coincides with recognized risk factors (previous preeclampsia or FGR, chronic hypertension, IVF conception, nulliparity, age over 40 years, or an interpregnancy interval over 10 years) or with features that plausibly increase placental risk (diffuse disease, an enlarged uterus, previous adenomyomectomy) at a guideline dose and schedule (81–150 mg daily, started at 12 weeks and in any case before 16 weeks, and continued until delivery as recommended by ACOG and NICE, rather than stopped at 36 weeks as in the ASPRE protocol). Doses above 150 mg are not suggested. Compared with 81 mg, 162 mg did not significantly reduce preeclampsia in two recent trials. In one of them, it was associated with lower birth weight and more placental abruptions (8 versus 0) [99,100]. Doses above 162 mg have not been evaluated [95], and abruption and hemorrhage are already more frequent in adenomyosis (Section 4.4 and Section 5.1). Aspirin prevents but does not treat established FGR, so serial growth scans and umbilical artery Doppler remain necessary (Section 5.2). A prospective trial of aspirin in pregnancies with adenomyosis is a research priority (Section 8).

## 5. Adenomyosis and Childbirth

### 5.1. Labor, Delivery, and Mode of Birth

Adenomyosis also affects labor and the mode of birth. In the Dutch ADENO study (7925 women with histologically confirmed adenomyosis and over 4.6 million controls), it was associated with hypertensive disorders (aOR 1.37), preeclampsia (aOR 1.37), emergency cesarean delivery (aOR 1.54), failure to progress (aOR 1.24), retained placenta (aOR 1.29), and postpartum hemorrhage (aOR 1.23), but not with operative vaginal delivery or oxytocin augmentation [34]. These findings are compatible with impaired myometrial contractility in labor and the third stage, though not proof of it.

In a Japanese registry, the emergency cesarean rate was 20.9% with adenomyosis versus 8.3% without, and adenomyosis independently predicted placenta accreta (OR 2.26) [33]. Pooled data confirm higher odds of cesarean delivery (OR 2.48) and malpresentation (OR 3.05) [37], with the latter perhaps reflecting an asymmetrical, enlarged uterine cavity [87].

**Abnormal uterine contractility: a plausible but unproven mechanism.** Adenomyosis alters myometrial function before pregnancy. Imaging shows hyperperistalsis and dysperistalsis of the junctional zone [6,9], and symptomatic women show increased contractility and oxytocin receptor overexpression that correlate with dysmenorrhea severity [101]. In pregnancy, hypertrophic, disorganized muscle interrupted by ectopic glands might contract less efficiently and less synchronously, which would explain slow or arrested labor, poor retraction after delivery, and atony, consistent with the excess of failure to progress, emergency cesarean delivery, retained placenta, and postpartum hemorrhage in ADENO [34]. In a prospective matched cohort of women with endometriosis and/or adenomyosis, postpartum hemorrhage occurred in 13% versus 1%, cesarean delivery in 40% versus 26%, and spontaneous vaginal birth in 43% versus 67%. Hemorrhages were due to atony, placenta accreta, myometrial hypervascularization, and intraoperative tears, with prolonged labor contributing [102]. Because the cohort combined both diseases and the estimates remained imprecise after limited adjustment, it does not isolate the effect of adenomyosis. Induction rates were similar [102], and oxytocin augmentation was not increased [34]. Thus, whether dysfunctional labor reflects weak or discoordinated contractions is unclear. Uterine activity in labor has never been recorded in adenomyosis, and the contractility hypothesis remains a plausible explanation rather than an established finding (Table 4).

Uterine rupture is rare (0.2% of pregnancies with adenomyosis versus 0.01% without [33]), and the largest meta-analysis, to which this registry [33] was the only contributor, found no significant difference [78]. The concern is greater after adenomyomectomy, where the scar is often extensive [103] (Section 5.3).

In practice, adenomyosis alone is not an indication for cesarean delivery, and vaginal birth can be planned in most women. However, units should anticipate slow progress, abnormal placentation, retained placenta, and postpartum hemorrhage. After uterus-sparing surgery for adenomyosis, planned cesarean delivery is widely recommended (Section 5.2 and Section 7.1).

### 5.2. Indications and Timing of Cesarean Delivery

No guideline addresses delivery in adenomyosis. In uncomplicated pregnancies, it is not an indication for cesarean or early delivery, and the mode and timing of delivery are determined by any associated complication (Table 5).

For placenta previa, ACOG suggests delivery at 36+0–37+6 weeks (weeks+days) [104], and the 2026 Royal College of Obstetricians and Gynaecologists (RCOG) guideline suggests planned birth no later than 37+6 weeks [105]. For suspected PAS, ACOG and the Society for Maternal-Fetal Medicine (SMFM) suggest planned cesarean delivery at 34+0–35+6 weeks in a specialized center [106], whereas RCOG, as a good practice point, suggests 36+1–37+0 weeks when PAS is highly probable and there is no bleeding or preterm risk [105]. Because adenomyosis increases both risks, placental site and invasion should be assessed at the second-trimester scan.

For a small fetus, timing depends on severity and umbilical artery Doppler findings. Isolated FGR with an estimated fetal weight (EFW) between the third and tenth percentiles and normal Doppler findings can be delivered at 38+0–39+0 weeks and at 37+0 weeks when below the third percentile [107]. SMFM advises 37 weeks with decreased diastolic flow, 33–34 weeks with absent and 30–32 weeks with reversed end-diastolic velocity, considering cesarean delivery in the last two [108]. FGR alone is not an indication for cesarean delivery [107].

After adenomyomectomy, planned cesarean delivery is widely recommended, although no guideline specifies its timing, which should be individualized rather than fixed (Section 7.1). Rupture has been reported in 2.8–12.5% of women in small series at 16–35 weeks and, in a large series, exclusively after surgery that had entered the cavity. Most authors advise delivery at 35–36 weeks [109]; a residual myometrial thickness of 7 mm or less was associated with an abnormal course in 60% of cases [110]. Among 22 deliveries after adenomyomectomy, all by cesarean at a mean of 36.2 weeks, one rupture occurred at 27 weeks [111]. ACOG suggests 37+0–38+6 weeks after myomectomy or earlier when the surgery was extensive or complicated [104]. The choice between 35 and 38 weeks therefore depends on the extent of the excision, entry into the cavity, residual myometrial thickness, placental site and symptoms, and on balancing the risk of rupture against late-preterm morbidity, and should be made for each woman in a center with neonatal care (Table 5).

**Table 5:** Suggested timing and mode of delivery in pregnancies with adenomyosis.

Clinical Situation	Suggested Timing of Delivery	Mode of Delivery	Reference
Adenomyosis without complications	Term	Vaginal birth can be planned; cesarean for obstetric indications	[34,89]
Placenta previa	36+0–37+6 weeks (ACOG); no later than 37+6 weeks (RCOG)	Planned cesarean	[104,105]
Suspected placenta accreta spectrum	34+0–35+6 weeks (ACOG/SMFM); 36+1–37+0 weeks (RCOG, good practice point, if no bleeding or preterm risk)	Planned cesarean in a specialized center, often with hysterectomy	[105,106]
FGR, EFW 3rd–10th percentile, normal Doppler	38+0–39+0 weeks	Vaginal birth possible	[107,108]
FGR, EFW below 3rd percentile or decreased diastolic flow	37+0 weeks	Vaginal birth possible	[107,108]
FGR with absent end-diastolic velocity	33–34 weeks	Consider cesarean	[108]
FGR with reversed end-diastolic velocity	30–32 weeks	Consider cesarean	[108]
Previous adenomyomectomy	Individualized, usually 35–38 weeks, according to the extent of surgery, entry into the cavity, residual myometrial thickness and placental site (timing not specified by guidelines)	Planned cesarean	[104,109,111]

ACOG, American College of Obstetricians and Gynecologists; EFW, estimated fetal weight; FGR, fetal growth restriction; RCOG, Royal College of Obstetricians and Gynaecologists; SMFM, Society for Maternal-Fetal Medicine.

### 5.3. Uterine Rupture in Pregnancy and Labor

Rupture of an unscarred uterus can occur, most often before labor. A review collected 15 adenomyosis-related ruptures occurring from 18 weeks to term, including a primigravida with spontaneous fundal rupture at 22 weeks, fetal death, and hysterectomy, with transmural adenomyosis at the rupture site [112]. Rupture at term in a primigravida who presented in cardiac arrest, with diffuse adenomyosis at the posterior rupture site [113], and rupture during induced labor at 39 weeks [114] have also been reported. The proposed mechanism is weakening of the myometrium by transmural adenomyosis, with decidualized ectopic stroma separating and thinning the muscle fibers [112].

At the population level, rupture occurred in 0.2% versus 0.01% of pregnancies in a Japanese registry [33], and the largest meta-analysis, to which only this registry contributed, found no significant difference (very low certainty) [78]. The absolute risk in unoperated adenomyosis is therefore low. The risk is higher after uterus-sparing surgery, although the denominators are small. Among 304 pregnancies after adenomyomectomy, five ruptures (1.6% of pregnancies, 2.0% of women) occurred at 16–31 weeks, all before labor and all with the placenta overlying the scar, with two fetal deaths and one hysterectomy [115]. A review of 2365 adenomyomectomies recorded 13 ruptures in 363 deliveries (3.6%) [116]. One rupture occurred among 18 pregnancies in another cohort [92]. Three third-trimester ruptures occurred among 34 deliveries (8.8%) after cold-knife adenomyomectomy [117]. A pooled series from small studies reported rates of up to 12.5% per patient [109]. After enucleation of adenomyosis and conception by intracytoplasmic sperm injection (ICSI), a rupture at 25 weeks expelled the fetus into the abdominal cavity, although the infant survived [118]. Previous uterine surgery, rather than adenomyosis itself, is thus the main determinant of rupture risk, and the two situations call for different counseling (Table 6).

Sudden abdominal pain, an abnormal fetal heart rate, or hemodynamic instability in a pregnant woman with adenomyosis should therefore prompt consideration of rupture, even without prior surgery or labor (Table 6).

## 6. Adenomyosis and the Newborn

### 6.1. Neonatal Outcomes

Newborns of women with adenomyosis are about twice as likely to be SGA or of low birth weight and more than three times as likely to be born extremely preterm. ORs of 2.86 for SGA and 2.82 for low birth weight were reported in the earlier meta-analysis [37]. RRs of 1.79, 2.29, and 3.71 for SGA, low birth weight, and extreme preterm birth, respectively, were reported in ADELIN [78], and smaller but consistent associations (SGA, RR 1.33; low birth weight, RR 2.04) were reported in a 2026 synthesis [85] (Figure 10). In absolute terms, delivery before 32 weeks occurred in 7.7% versus 1.7% of IVF pregnancies [40], and FGR occurred in 5.3% versus 3.8% of pregnancies in the Japanese registry [33].

In the largest analysis, FGR, neonatal intensive care admission and uterine rupture did not differ [78], and data on stillbirth, neonatal mortality, and long-term development, including bone health (Section 6.3), are too scarce to allow firm conclusions.

### 6.2. Neonatal Respiratory Morbidity

No study has reported neonatal respiratory distress syndrome (RDS), transient tachypnea, or the need for respiratory support as an outcome of adenomyosis, and the largest meta-analysis found no difference in neonatal intensive care admission [78]. Any increased risk is therefore inferred, not demonstrated, through three pathways: prematurity because extreme preterm birth is more than three times as frequent [78] and delivery before 32 weeks occurred in 7.7% versus 1.7% of pregnancies after IVF [40]; planned early delivery because placenta previa and PAS lead to cesarean delivery at 34–37 weeks [104,105,106] and prelabor cesarean delivery increases respiratory morbidity even at term [119]; and intrauterine infection and placental dysfunction because chorioamnionitis was more frequent in pregnancy-related hysterectomies with adenomyosis [94] and PPROM and FGR are recognized risk factors. Of these, only the higher rates of preterm birth and indicated early delivery are established in adenomyosis; the respiratory consequences are those of prematurity and early cesarean delivery in general.

Management follows the guidelines that apply to any pregnancy at risk of preterm birth [120,121,122]. When delivery is expected between 24+0 and 33+6 weeks, a course of antenatal corticosteroids (betamethasone 12 mg intramuscularly in two doses 24 h apart, or dexamethasone 6 mg in four doses 12 h apart) is recommended. In the late-preterm period (34+0–36+6 weeks), a course may be offered to women without pregestational diabetes who have not received a previous course, as in the Antenatal Late Preterm Steroids (ALPS) trial. Before 37 weeks, a course reduces RDS, perinatal death, and intraventricular hemorrhage [121]. In ALPS, betamethasone reduced the composite of respiratory support, stillbirth, or neonatal death from 14.4% to 11.6%, at the cost of more neonatal hypoglycemia [122]. In the absence of an obstetric indication, planned cesarean delivery should not be scheduled before 39 weeks, and the neonatal team should be informed of the maternal diagnosis and of any placental complication; none of these measures is specific to adenomyosis.

### 6.3. Bone Health: Metabolic Bone Disease of Prematurity and Maternal Vitamin D

No study has examined bone health in the children of mothers with adenomyosis; the risk discussed here is inferred from the excess of extreme prematurity, low birth weight, and FGR. About 80% of fetal bone mineral is accrued during the third trimester, which preterm infants largely miss, and placental insufficiency alters placental phosphate transport. Thus, these infants are at risk of metabolic bone disease of prematurity, a usually silent condition that in its severe forms causes fragility fractures and reduced growth and is followed by a lower peak bone mass in adulthood [123,124]. Screening is recommended for very-low-birth-weight or very preterm infants regardless of the maternal condition [123,124]; any additional risk in the offspring of women with adenomyosis is a research question, not an established outcome.

**Maternal vitamin D deficiency.** Serum 25-hydroxyvitamin D [25(OH)D] is lower in women with endometriosis [125] and adenomyosis, with deficiency (<50 nmol/L) in 74.7% versus 28.6% of women in a propensity-matched study [126]. The lowest levels were noted in those with coexistent deep endometriosis [127]. The fetus depends on maternal vitamin D, and maternal deficiency is linked to neonatal hypocalcemia and congenital rickets [128]. Adequate maternal levels have been associated with higher bone mineral content in the offspring in several, though not all, cohorts [129]. Whether maternal deficiency adds to the bone risk of preterm infants of mothers with adenomyosis has not been studied. Treatment of a documented deficiency is standard care in any pregnancy, but ACOG does not recommend routine screening of all pregnant women, reserving measurement of 25(OH)D for women at increased risk of deficiency [130]. Screening specifically for adenomyosis is an untested hypothesis, not a recommendation.

**Birth-related fractures.** No fetal or neonatal fracture attributed to maternal adenomyosis has been reported, including among the ruptures in Section 5.3. Birth-related fractures are rare (about 0.23 long-bone fractures per 1000 live births) and are associated with emergency cesarean delivery, prematurity, malpresentation, abnormal lie, multiple pregnancy, and difficult extraction [131,132,133], several of which are more frequent in adenomyosis. A higher risk is conceivable but unproven. Careful extraction through an adequate incision is advisable when the uterine wall is thickened, and this outcome should be recorded in future cohorts.

### 6.4. Interpretation

Most of the neonatal burden is probably mediated by the obstetric complications described in Section 4 and Section 5 (Figure 11). Preterm birth, whether spontaneous, after PPROM or indicated for preeclampsia, exposes the neonate to the morbidity of prematurity, and placental insufficiency explains the excess of SGA and low birth weight. Whether adenomyosis has any direct effect on the fetus is unknown, and the respiratory and skeletal consequences discussed above are those of prematurity and placental dysfunction, not demonstrated effects of adenomyosis itself.

Interpretation is limited by confounding. Women with adenomyosis are older, more often conceive through ART, and frequently have endometriosis or fibroids, each associated with adverse outcomes. Few studies adjust for all of them, and adenomyosis is often diagnosed before or after rather than during pregnancy. These limitations are reflected in the low to very low certainty assigned by recent syntheses [78].

## 7. Management and Clinical Implications

No randomized trial has shown that any intervention improves live birth in adenomyosis, so management rests on individualized counseling and observational evidence.

### 7.1. Conservative Surgery

In women who wish to have children, treatment should be conservative (uterus-sparing). Hysterectomy precludes pregnancy, so every option should preserve the uterus and its capacity to carry a pregnancy. Uterus-sparing surgery removes as much adenomyotic tissue as possible while preserving a myometrium able to sustain a pregnancy. It is reserved for symptomatic women who wish to conceive and in whom medical therapy has failed, particularly those aged 39 years or younger with adequate ovarian reserve, focal disease, and repeated IVF failure [134,135]. Because of its obstetric risks, it should be performed in expert centers after hormonal therapy has been considered [136]. Complete excision is feasible mainly for focal disease (adenomyoma), whereas diffuse disease allows only cytoreduction. Focal and external adenomyosis are considered separately below, and diffuse disease is discussed together with the surgical approach, comparative studies, and large series.

**Focal adenomyosis (adenomyoma).** A focal lesion is best suited to complete excision, and guidelines favor a minimally invasive route [137,138]. Unlike a fibroid, an adenomyoma has no cleavage plane, so excision relies on visual and tactile judgment. In 105 laparoscopic adenomyomectomies with transient uterine artery occlusion and three-layer closure, there were no major complications. Dysmenorrhea resolved in 93.0% of women and menorrhagia in 76.9%. Recurrence was 10.5%, and lesions larger than about 5 cm were considered less suited to laparoscopy [139]. Reproductive results exceed those in diffuse disease (pooled pregnancy and delivery rates of 58.3% and 46.2% versus 47.3% and 37.4%) [140], with similar miscarriage but more preterm birth (10.9% versus 4.5%) in an earlier review [141]. In total, 41.1% of women attempting natural conception after excision followed by six courses of GnRHa conceived within 3 years [31]. Whether excision improves on medical treatment or ART alone is uncertain. In a Japanese nationwide survey, pregnancy rates in focal disease were similar after surgery (39.1%), without pretreatment (43.0%), and after medical pretreatment (48.0%), although no pregnancy after surgery ended in miscarriage [30]. Seven of 10 women with focal posterior wall lesions managed without surgery conceived, with surgery reserved for long-term ART failure, uncontrolled pain, or a thin luteal-phase endometrium [142]. A 2026 systematic review of 43 studies confirmed that laparoscopy is used mainly for focal disease, with less blood loss. However, unlike earlier reviews, the study found higher pregnancy and live birth rates after surgery for diffuse disease and similar rupture rates [143]. Cystic adenomyoma, a special focal form, was treated with uterine preservation in 18 women, with minor complications and seven successful pregnancies [144]. Laparoscopic excision is not free of obstetric risk. A review of 2365 adenomyomectomies recorded 13 ruptures in 363 deliveries (3.6%), estimated the rupture risk at over 1.0% versus 0.26% after myomectomy, and noted an apparently higher risk after laparoscopic than after open adenomyomectomy for focal disease [116].

**External adenomyosis.** External (extrinsic) adenomyosis, or focal adenomyosis of the outer myometrium, arises on the serosal side, usually posteriorly; spares the junctional zone; and is closely linked to posterior deep endometriosis [8,14,16]. It was present on MRI in 56.5% of women operated on for deep endometriosis and associated with more deep lesions [145]. It is therefore usually excised from the serosal side during surgery for deep endometriosis, and no series is restricted to this phenotype. Leaving it in place appears costly. When it was not excised during surgery for rectovaginal and colorectal endometriosis, 11.9% of women with adenomyosis conceived versus 43.0% of those without (RR 0.32) [146]. After colorectal resection, dysmenorrhea improved only in women without adenomyosis or whose adenomyosis was excised [147]. After rectovaginal shaving, dyspareunia improved less when adenomyosis coexisted [148]. When it was excised together with the rectovaginal nodule, clinical pregnancy was 36.0% versus 42.6% in women without adenomyosis, with no rupture [149]. However, posterior wall involvement lowered the chance of pregnancy after laparoscopic adenomyomectomy (OR 0.18) [135]. The surgery is demanding. Coexisting adenomyosis independently predicted complications of laparoscopy for deep endometriosis (33.8% versus 12.5%) [150]. Rupture of the lower posterior wall in labor has been reported after resection of rectovaginal endometriosis [151]. Hysterectomy for adenomyosis was the most frequent repeat operation (4.1%) among 1092 women followed after complete excision of endometriosis [152]. These small non-randomized studies rarely classified the phenotype, so the benefit of excising external adenomyosis remains unconfirmed.

**Perioperative outcomes and surgical approach.** Adenomyomectomy is major surgery, with a median blood loss of 500–750 mL and transfusion in 40–68% of infertile women [60]. In diffuse disease, a mean operating time of 321 min, a mean blood loss of 507 mL, and complications in 8.9% of cases were reported [153], although serious complications are uncommon (hysterectomy 1.2%, serious complications 0.2% of 1843 operated women) [154]. The approach depends on disease extent. Purely laparoscopic surgery for smaller lesions had the lowest blood loss and shortest stay, with laparoscopic-assisted and open surgery being comparable [155]. Laparotomy, used for larger and mostly diffuse lesions, took longer with greater blood loss and was recommended for diffuse disease. Laparoscopy was recommended for focal lesions under 5 cm [156]. Robot-assisted surgery took longer than open surgery but required fewer transfusions and a shorter stay [157]. A 2026 meta-analysis of 22 retrospective studies in diffuse disease found no differences between laparoscopy and laparotomy in operating time, blood loss, complications, or reproductive outcomes, but more resected tissue and less recurrence were noted after laparotomy [158]. Temporary occlusion of the uterine arteries and utero-ovarian vessels reduced blood loss from 344 to 112 mL without affecting AMH [159]. Mean loss was 207 mL in 116 laparotomies with transient occlusion, although 44.8% received red cell transfusions [160]. Intrauterine adhesions were found in only three of 137 women after double-flap surgery, in which the cavity is opened [161].

**Symptom control and recurrence.** Menorrhagia was controlled in 68.8% of women after complete and 50.0% after partial excision in a review of 64 studies [162]. A review of 19 studies confirmed improvement in pain, menorrhagia, and uterine volume with both approaches [163]. Dysmenorrhea is reduced in 54.6–84.6% of women across techniques [164]. After asymmetric dissection, a cytoreductive technique for diffuse disease, the dysmenorrhea score fell from 9.4 to 0.8 and anemia improved in all 44 women [165]. An earlier review reported relief in over three-quarters of women [103]. Recurrence is common. In 133 women followed for a mean of 52 months, it reached 39.1%. It was more frequent with posterior wall involvement, two or more lesions, laparotomy, and endometriosis and less frequent with postoperative progestogens or GnRHa [166].

**Fertility outcomes.** Pregnancy rates were 60.5% after complete excision and 46.9% after partial excision in the 2014 review [162]. In a 2025 meta-analysis (903 women), pregnancy after adenomyomectomy was 50.1%, delivery 39.5%, and miscarriage 16.3%. In total, 40.5% of conceptions required IVF, and 99.6% of births were by cesarean [140]. Outcomes are better in focal than in diffuse disease (pregnancy 52.7% versus 34.1% [141]; live birth 55.6% versus 29.4% [109]), and age is decisive, with clinical pregnancy in 41.3% at 39 years or younger and 3.7% at 40 or older [135]. In the triple-flap series, in which the uterine wall is reconstructed by laparotomy with overlapping myometrial flaps after radical excision, 16 of 26 women who wished to conceive became pregnant, and 14 delivered at term [167] (Table 7).

**Obstetric safety.** Rupture after adenomyomectomy has been reported in 2.8–12.5% of patients in small series at 16–35 weeks and, in a large series, exclusively after surgery that had entered the cavity [109]; in 6.8% after surgery for diffuse disease and none after focal disease in one review [141]; in 13 of 363 deliveries (3.6%) in a worldwide review [116]; and in three of 34 deliveries (8.8%) in a recent cohort [117] (Section 5.3). PAS complicated nine of 18 pregnancies after adenomyomectomy in one cohort [92]. A residual myometrial thickness above 10 mm has been proposed as a safety threshold because 60% of women with 7 mm or less had an abnormal course [109,110]. Suggested intervals before conception range from 3 months [134] to 12–24 months [109], and planned cesarean delivery is recommended, with the timing individualized between 35 and 38 weeks according to the extent of surgery, residual myometrial thickness, and placental site (Section 5.2).

**Comparative studies.** No randomized trial has compared conservative surgery with medical treatment or ART. In small retrospective series, the 3-year cumulative clinical pregnancy rate was 46.4% after conservative surgery with or without GnRHa versus 10.8% after GnRHa alone, with delivery in 32.1% versus 8.1% [168]. The rate was 44.4% versus 13.6% in a second series in which all conceptions were spontaneous [169]. Pooled spontaneous pregnancy was 40.7% with 24 weeks of GnRHa after surgery versus 15.0% without [170]. Larger data sets are less favorable. In the Japanese nationwide survey (535 infertile women), pregnancy rates were 41.6% after surgery, 43.5% after medical pretreatment, and 41.3% without either, with similar miscarriage rates and no significant difference with ART [30]. Among 221 infertile women with diffuse disease, cold-knife adenomyomectomy before IVF/ICSI reduced miscarriage compared with GnRHa alone (RR 0.18) without increasing clinical pregnancy or live birth, with a cumulative live birth rate of 41.0% in operated women who completed a transfer [117]. All these comparisons are non-randomized and confounded by indication, and the reported pregnancy rates vary with surgical technique, patient selection, disease extent, and postoperative management. The available evidence therefore does not establish that surgery is superior to medical management or ART, and the decision must weigh the chance of pregnancy against the obstetric risks of an operated uterus, above all rupture and abnormal placentation (see “Pregnancy after surgery” below).

**Large series and predictors of pregnancy.** In a single-center cohort of 322 women treated by uterus-sparing surgery, 72.1% of those who attempted pregnancy conceived, with 59 live births, and the cumulative 5-year live birth rate was 79% for localized versus 37% for diffuse disease, which independently predicted a lower chance of live birth (hazard ratio 0.4) [171]. After double-flap adenomyomectomy for diffuse disease, followed by GnRHa and at least 1 year of waiting, 40.9% of 137 women conceived, with 45 live births, 22.6% miscarriage, and no rupture. The 3-year cumulative pregnancy rate was 70.1% when the postoperative junctional zone measured 8.5 mm or less versus 20.9% when thicker [161]. After wedge resection for severe disease, 30% of 70 women attempting pregnancy conceived, and 16 had a full-term birth [32]. In diffuse disease with previous IVF failure, 18 of 33 women conceived, and 10 had a viable pregnancy. All delivered by cesarean without rupture [153]. Pregnancy and delivery rates after adenomyomectomy (75.0% and 46.4%) did not differ significantly from those after intramural myomectomy (96.0% and 70.8%) [172]. In the laparoscopic series cited above for age, previous IVF predicted pregnancy (OR 6.22) and posterior wall involvement predicted a lower chance (OR 0.18), with 60.8% of women aged 39 or younger with previous IVF failure conceiving [135].

**Adjuvant hormonal therapy.** In a meta-analysis of 42 single-arm studies (5877 patients), recurrence after adenomyomectomy was 12.6%, and reintervention was 2.6%, with recurrence in 8.0% with perioperative or postoperative hormonal therapy versus 16.6% without [173]. In a prospective non-randomized study, symptoms relapsed within 2 years in 28.1% after six postoperative courses of GnRHa versus 49.0% after surgery alone [174]. For women postponing conception, maintenance dienogest after GnRHa (recurrence 4.6% versus 37.0%) [175] and a levonorgestrel intrauterine system placed at surgery (2.9% versus 32.8%) [176] reduced recurrence in retrospective cohorts, although both suppress fertility.

**Pregnancy after surgery.** Adenomyomectomy may change the profile of obstetric risk rather than add to it. Compared with unoperated adenomyosis, pregnancies after complete adenomyomectomy by laparotomy had less PPROM, preeclampsia, and SGA (0% versus 12–15%), with a similar preterm rate and more blood loss at cesarean [92]. After laparoscopic adenomyomectomy, FGR was less frequent (0 of 17 versus 7 of 26), but one uterus ruptured at 34 weeks at the site of a placenta percreta [177]. In a Japanese survey of 262 pregnant women with adenomyosis, the only rupture occurred among the 24 with previous surgery (4.2%) [178]. Even with an energy-free technique and imaging surveillance before transfer, three third-trimester ruptures occurred among 34 deliveries (8.8%) [117]. In the national survey of the Japan Society of Obstetrics and Gynecology, rupture occurred at 30–32 weeks after adenomyomectomy versus 37 weeks after previous cesarean delivery and 39 weeks in the unscarred uterus, with a poorer neonatal prognosis [179]. Rupture complicates 0.6% of pregnancies after myomectomy [180]. This is several-fold lower than the 2.0–12.5% after adenomyomectomy, although the comparison is indirect. Hysteroscopy after open adenomyomectomy showed endometrial defects in six of 10 women, which resolved within 7–21 months in five. The one pregnancy that occurred with a persisting defect was complicated by placenta percreta and rupture, so confirmation of endometrial restoration before conception has been proposed [181]. In pooled data, preterm delivery occurred in 18.4% of live births after adenomyomectomy, and adverse, mainly placental, outcomes were noted in 21.4% [140].

### 7.2. Pregnancy and Birth

Given the associations in Section 4, Section 5 and Section 6, women with known adenomyosis may reasonably be managed as a higher-risk obstetric population, with confirmation of the placental site, monitoring of cervical length, blood pressure and fetal growth, and birth planned in a unit able to manage postpartum hemorrhage and preterm neonates. Antenatal corticosteroids are given within the usual guideline windows when preterm or late-preterm delivery is anticipated (Section 6.2). Low-dose aspirin is an individualized decision based on the woman’s overall risk profile because adenomyosis alone is not a guideline indication (Section 4.5). Measurement of 25(OH)D or of homocysteine–folate status is not proposed as routine practice. Documented vitamin D deficiency is treated as in any pregnancy, folic acid supplementation is universal, and any role for adenomyosis-specific screening awaits prospective evaluation (Section 4.1 and Section 6.3). Single embryo transfer avoids the added risks of multiple pregnancy. These recommendations rest on expert opinion and extrapolation rather than on trials in women with adenomyosis (Figure 12).

## 8. Conclusions and Future Directions

The evidence reviewed can be summarized in three layers (Table 8). The established associations, consistent across meta-analyses and large cohorts although of low to very low certainty, are lower clinical pregnancy and live birth rates and a higher miscarriage rate after IVF. The excess miscarriage risk persists with donor oocytes, and the lower implantation and live birth rates are also seen after euploid embryo transfer. Other associations include higher rates of preeclampsia, preterm birth, PPROM, placental malposition and placenta accreta spectrum, cesarean delivery, dystocia, retained placenta, and postpartum hemorrhage, as well as more SGA, low-birth-weight, and extremely preterm newborns. In selected observational cohorts, pregnancy was reported in approximately half of infertile women after uterus-sparing surgery, with a risk of uterine rupture and abnormal placentation far higher than in unoperated adenomyosis. Proposed mechanisms, plausible but not demonstrated in women with adenomyosis, include defective remodeling of the junctional zone spiral arteries, reduced endometrial receptivity, abnormal myometrial contractility in labor, hyperhomocysteinemia and low folate, extra-ovarian AMH production, and, from an animal model of endometriosis-like disease, a shared developmental origin of the lesions and of a reduced follicle pool. Clinical measures fall into two groups: those supported by existing obstetric guidelines, applied when the corresponding complication or risk factor is present (timing of delivery for placenta previa, placenta accreta spectrum, and FGR; antenatal corticosteroids; aspirin for guideline-listed risk factors; universal folic acid; treatment of documented vitamin D deficiency), and proposals specific to adenomyosis that remain untested (aspirin for adenomyosis alone, screening for vitamin D or homocysteine–folate status, routine GnRHa pretreatment, surgery in preference to medical treatment or ART, and a fixed gestational age for delivery after adenomyomectomy).

Adenomyosis is common and underdiagnosed in women of reproductive age, with an average diagnostic delay of 11 years. TVUS with standardized MUSA terminology is the first-line test and MRI the second; no classification is universally accepted. The disease affects every stage of reproduction. After IVF, the deficit appears mainly uterine: oocyte number and quality are largely preserved, miscarriage remains increased with donor oocytes, and the hypotheses of extra-ovarian AMH production and of a primary ovarian impairment remain untested. In pregnancy, the risks are higher in diffuse disease and rise with uterine volume. Abnormal contractility is a plausible but unproven contributor to dysfunctional labor, and rupture of an unscarred uterus is possible but rare. Defective remodeling of the junctional zone spiral arteries is the leading hypothesis for much of this burden. In women who wish to conceive, treatment should be conservative. GnRHa pretreatment has not improved live birth. In selected observational cohorts, pregnancy was reported in approximately half of infertile women after uterus-sparing surgery, with better results in focal disease. Superiority over medical treatment or ART is unproven, and the operated uterus carries a risk of rupture and placenta accreta spectrum that warrants planned cesarean delivery at an individualized gestational age.

The certainty of this evidence is low to very low.

**Priorities for research include the following:**

- Universal use of the revised MUSA terminology and reporting of phenotype, location, and extent;
- Prospective, population-based pregnancy cohorts with imaging during or before pregnancy and measurement of maternal vitamin D and homocysteine–folate status;
- Adjustment for age, ART conception, endometriosis, and fibroids, including studies of ovarian reserve that test whether extra-ovarian tissue contributes to serum AMH;
- Randomized trials of GnRHa pretreatment, surgery, obstetric surveillance strategies, and low-dose aspirin, vitamin D, and folate supplementation, with live birth and neonatal outcomes as primary endpoints;
- Long-term follow-up of children born to mothers with adenomyosis.

Until such data are available, adenomyosis should be actively sought in women with subfertility, recurrent ART failure, or recurrent pregnancy loss; discussed openly in preconception counseling; and treated as a marker of higher obstetric and perinatal risk.

## Figures and Tables

**Figure 1:**
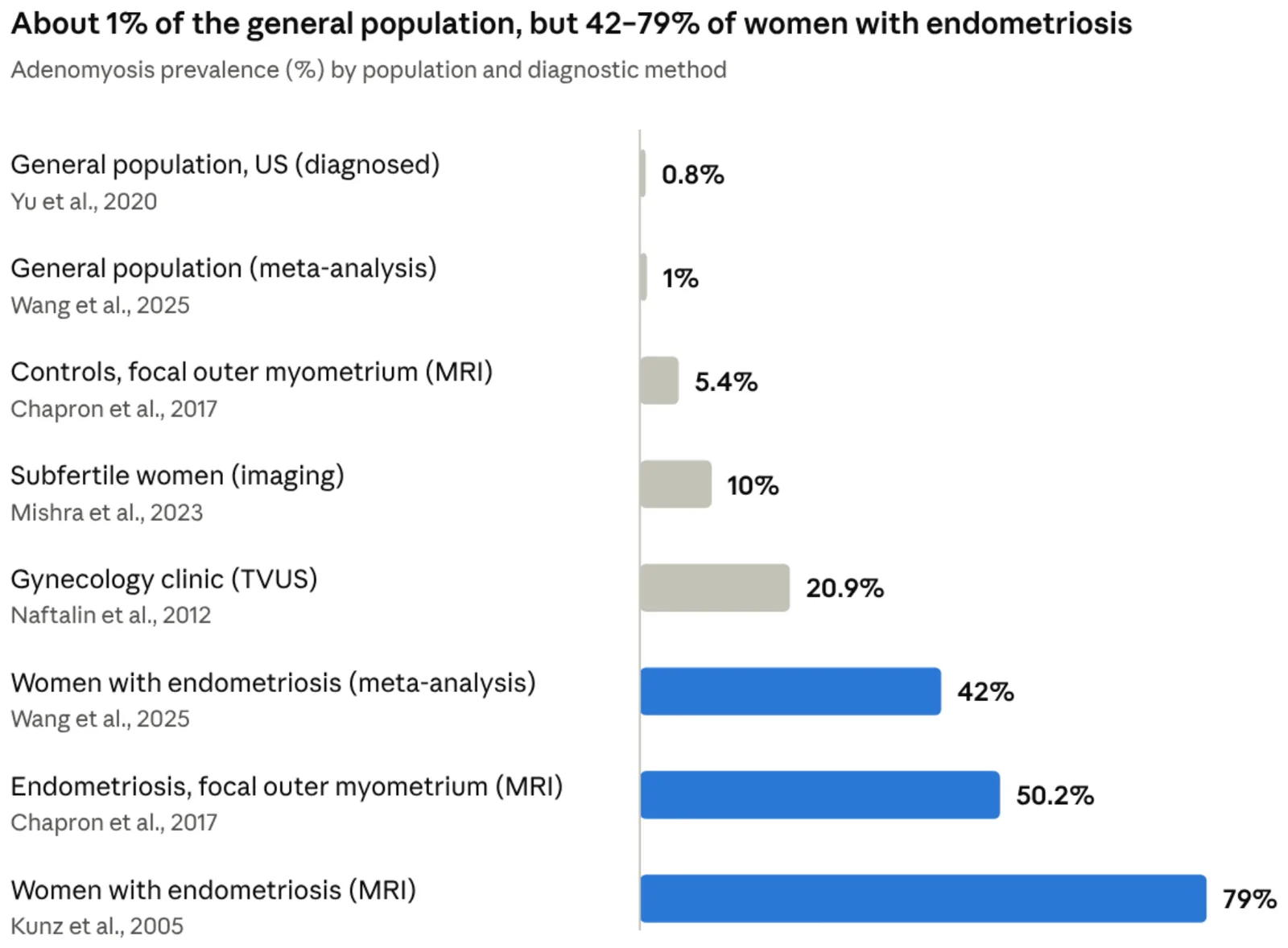
Prevalence of adenomyosis by population and diagnostic method. Data from Kunz et al. [6], Naftalin et al. [4], Yu et al. [3], Mishra et al. [5], Wang et al. [7] and Chapron et al. [8]. Blue bars, populations of women with endometriosis; gray bars, other populations.

**Figure 2:**
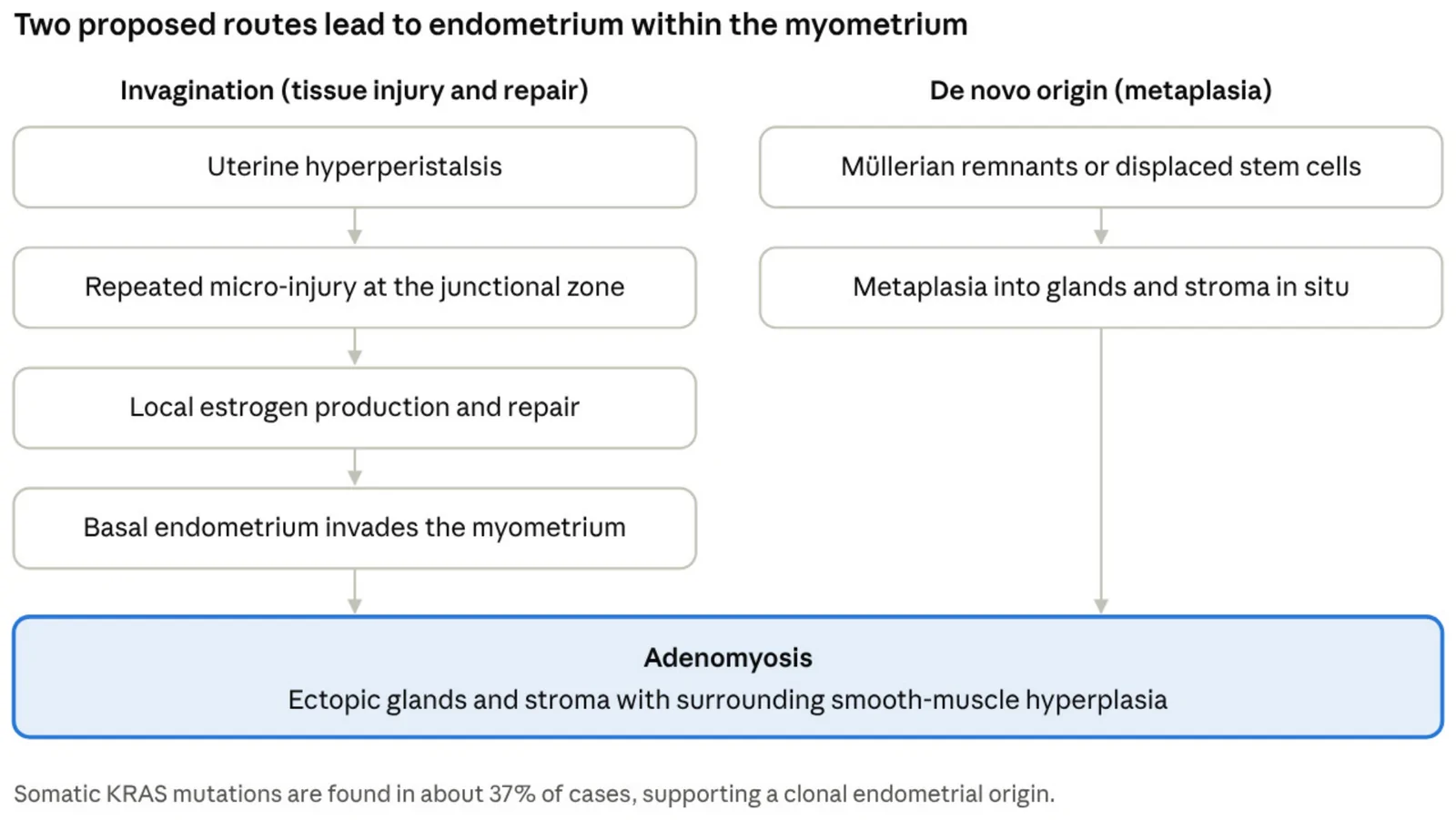
Main pathogenic hypotheses of adenomyosis. Original schematic based on Leyendecker, Wildt, and Mall [9] and Bulun et al. [10]; KRAS data from Inoue et al. [11]. Arrows indicate the proposed sequence of events in each route, and the blue box marks the common endpoint; the frequency of somatic KRAS mutations shown in the figure is from Bulun et al. [10] and Inoue et al. [11].

**Figure 3:**
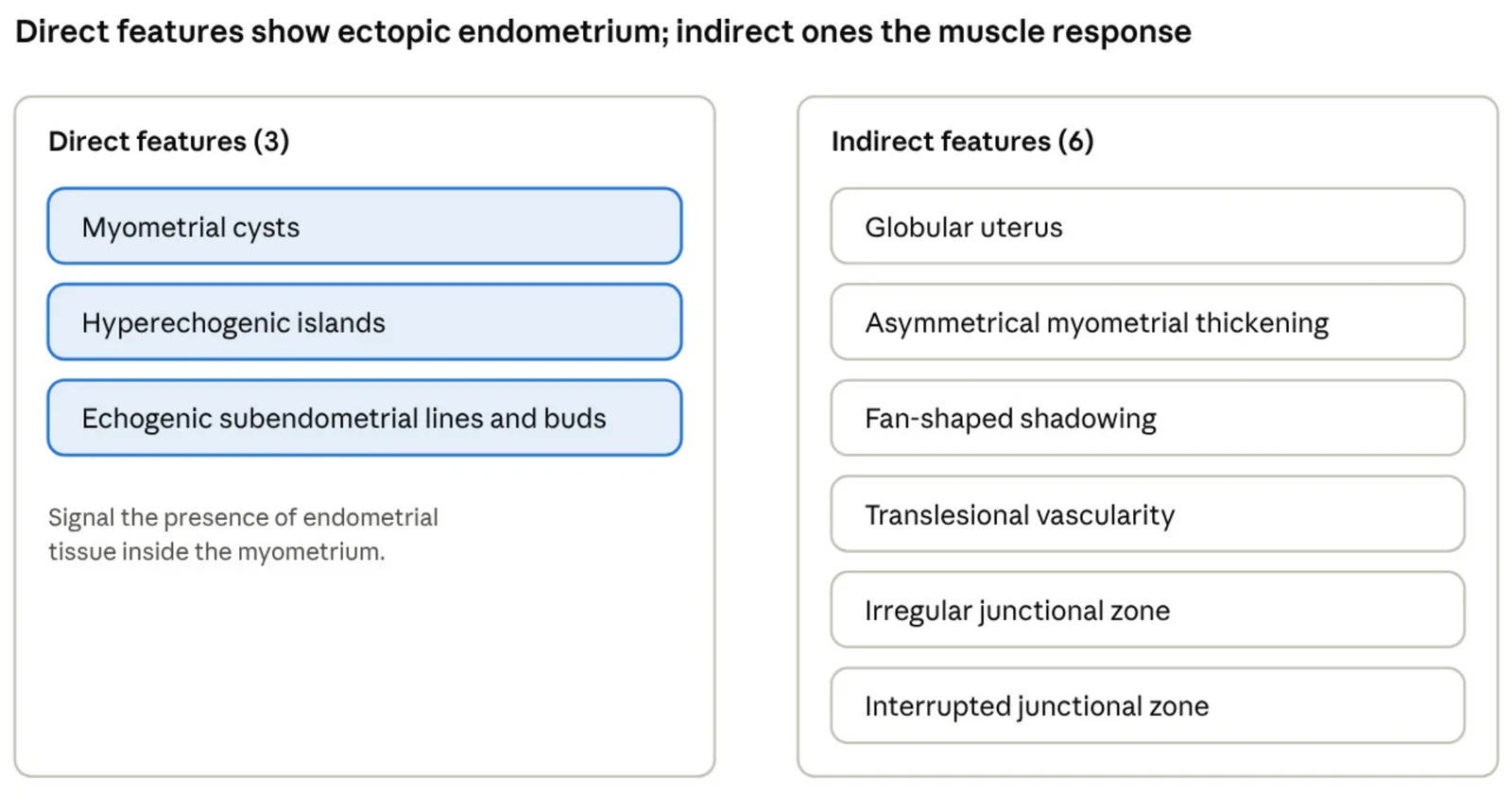
Sonographic features of adenomyosis according to the revised MUSA consensus. Original schematic based on Van den Bosch et al. [25] and Harmsen et al. [26].

**Figure 4:**
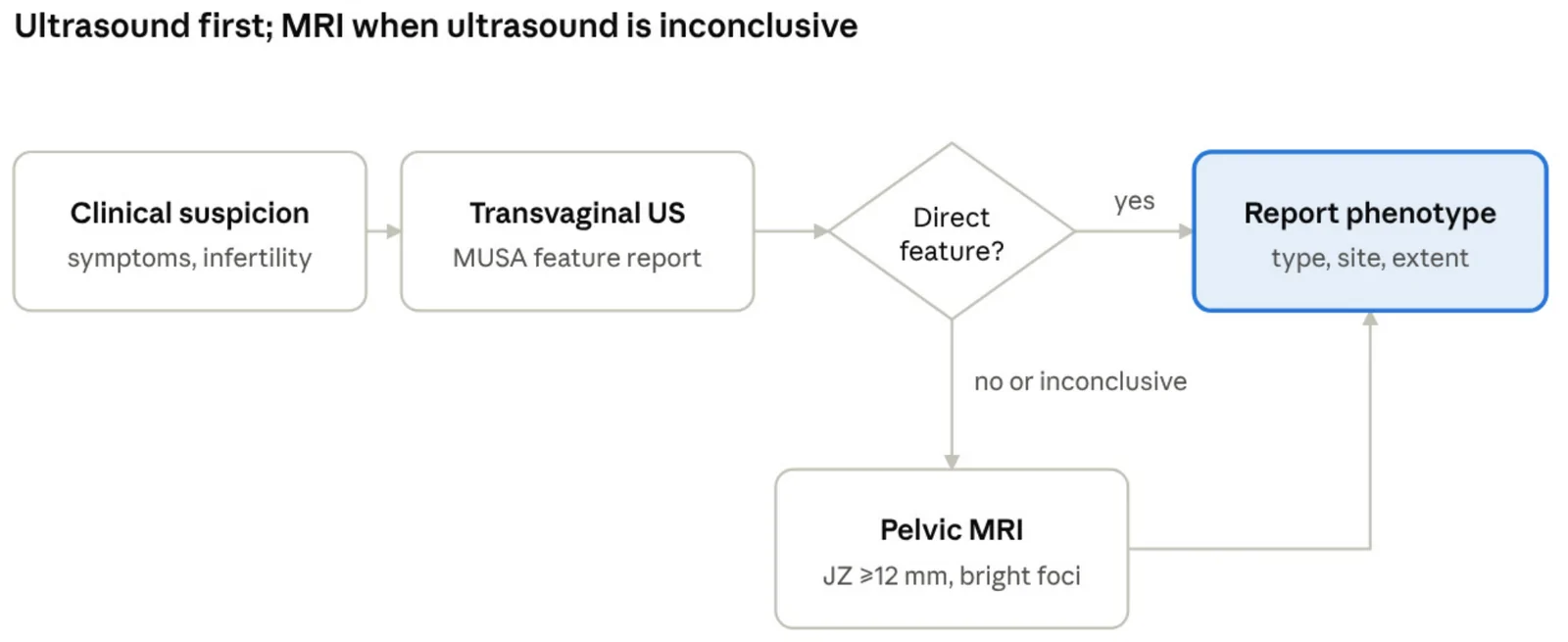
Integrated diagnostic pathway. Original schematic based on Chapron et al. [1]. Arrows indicate the sequence of steps, and the blue box marks the endpoint of the pathway (phenotype report). JZ, junctional zone; MRI, magnetic resonance imaging; MUSA, Morphological Uterus Sonographic Assessment; US, ultrasound.

**Figure 5:**
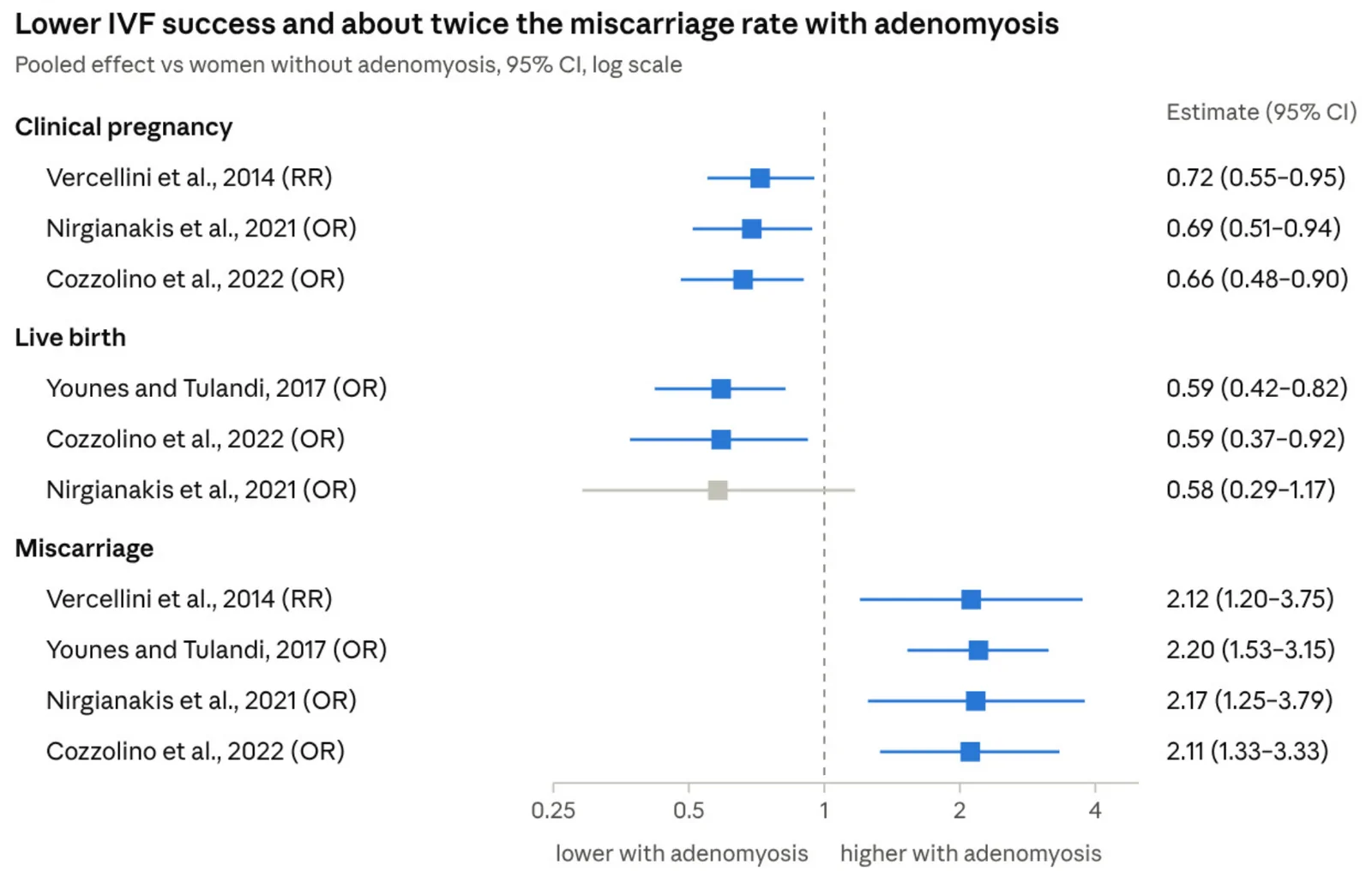
IVF/ICSI outcomes in women with versus without adenomyosis across four meta-analyses. Data from Vercellini et al. [35], Younes and Tulandi [36], Nirgianakis et al. [37], and Cozzolino et al. [38]; estimates are relative risks (RR) for Vercellini et al. and odds ratios (OR) for the other analyses, as labeled, with 95% confidence intervals (CI) on a logarithmic scale. Blue markers, 95% CI excludes 1; gray markers, 95% CI includes 1; the dashed vertical line marks the null value (1). ICSI, intracytoplasmic sperm injection.

**Figure 6:**
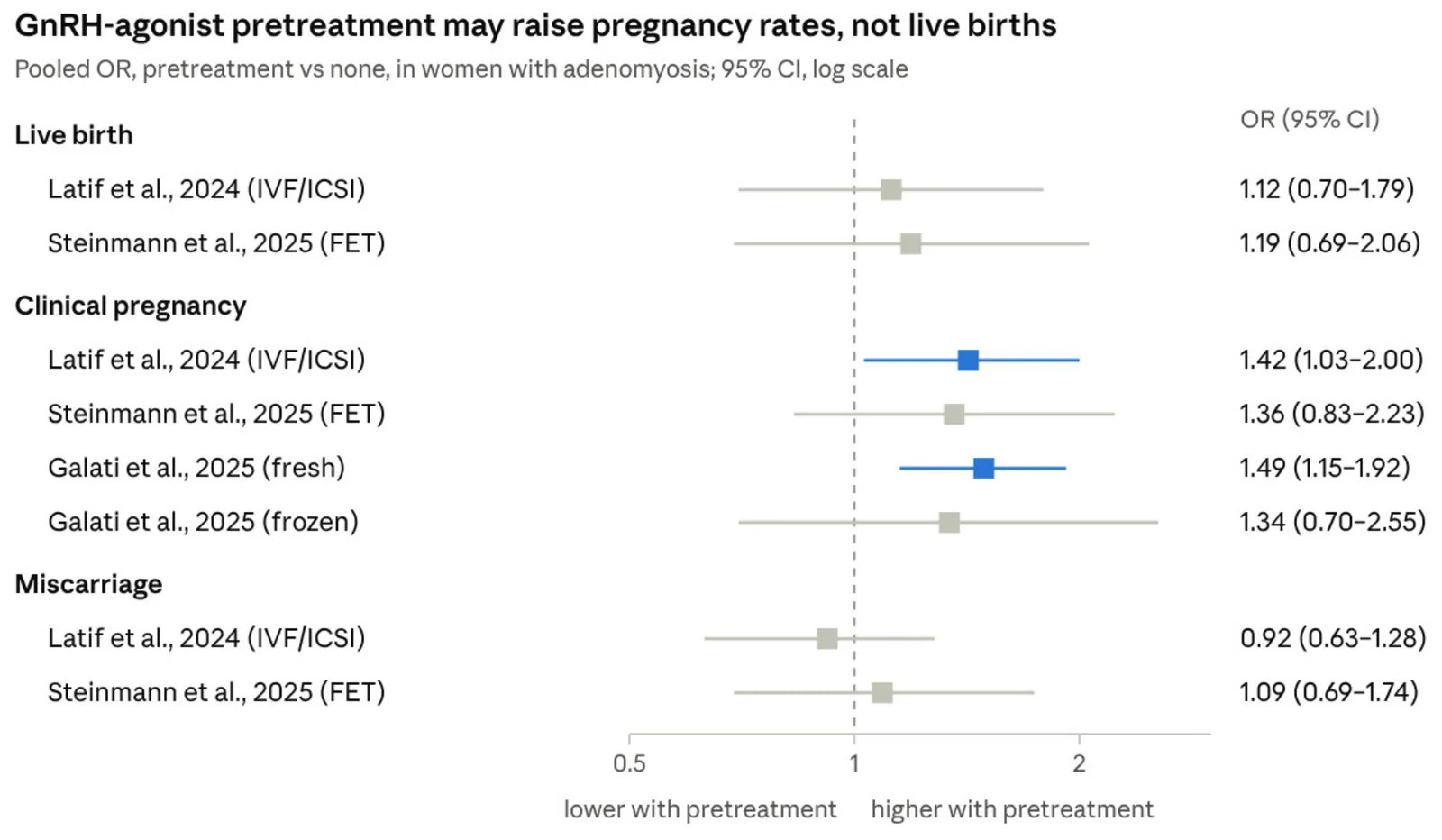
Effect of GnRHa pretreatment on ART outcomes in women with adenomyosis. Data from Latif et al. [47], Galati et al. [43], and Steinmann et al. [48]. Blue markers, 95% CI excludes 1; gray markers, 95% CI includes 1; the dashed vertical line marks the null value (1). ART, assisted reproductive technology; FET, frozen embryo transfer; GnRHa, gonadotropin-releasing hormone agonist; ICSI, intracytoplasmic sperm injection; IVF, in vitro fertilization; OR, odds ratio.

**Figure 7:**
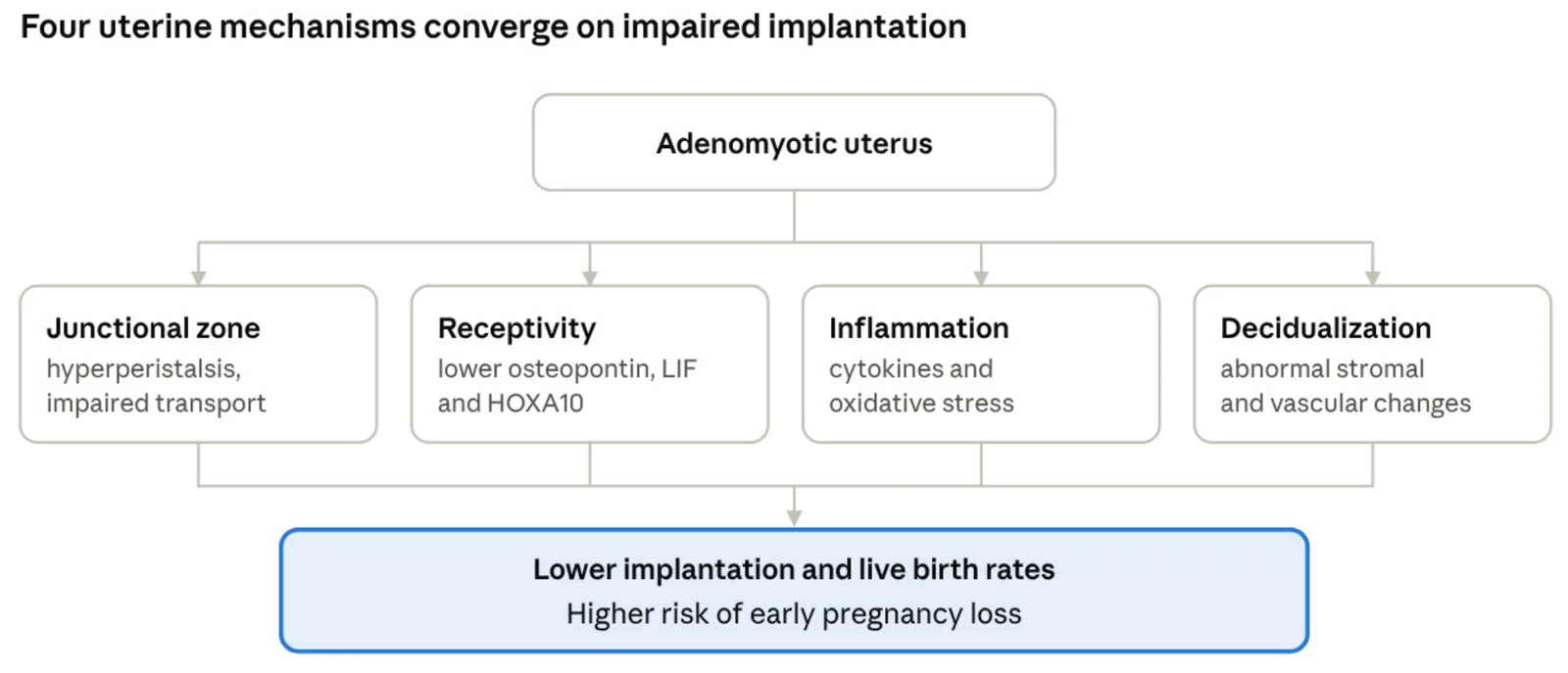
Proposed mechanisms linking adenomyosis to impaired implantation. Original schematic based on Kunz et al. [6]; Leyendecker, Wildt, and Mall [9]; and Brosens et al. [12].

**Figure 8:**
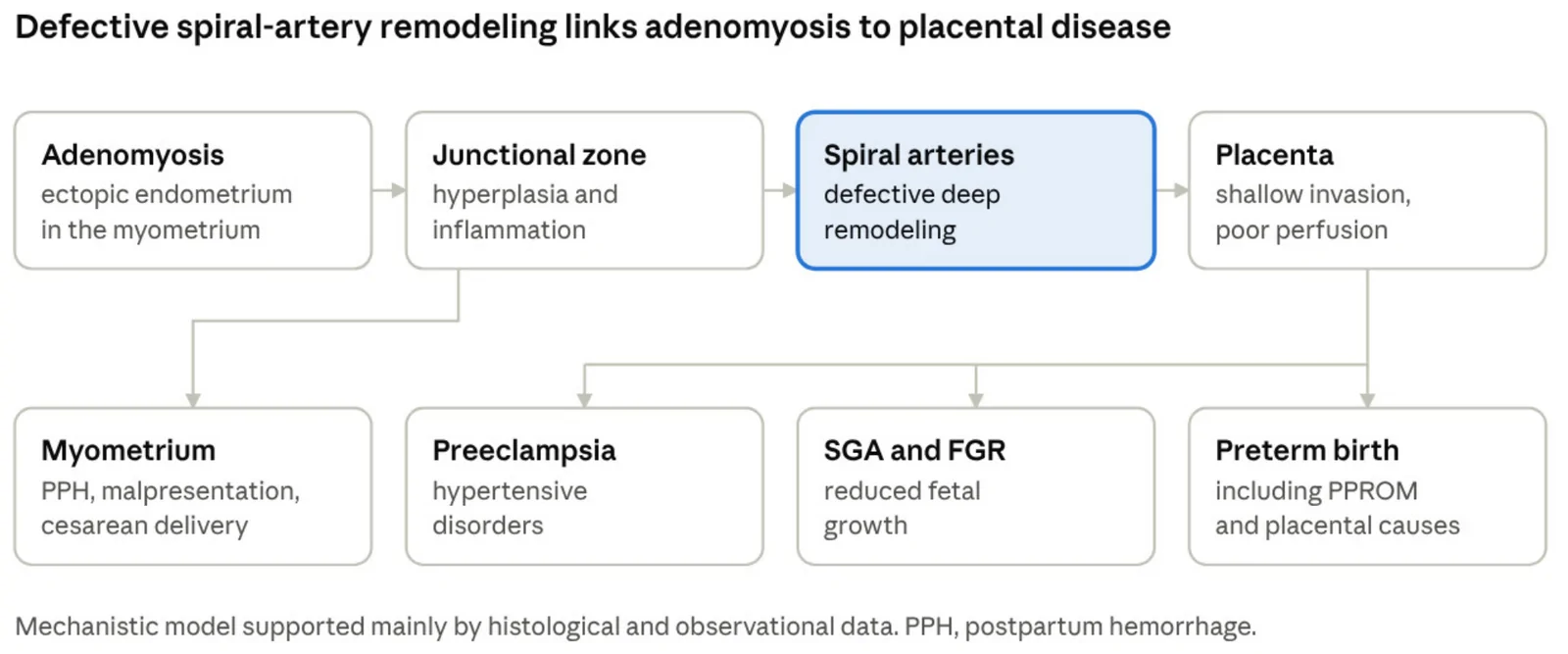
Proposed pathway from adenomyosis to the major obstetric syndromes. Original schematic based on Brosens et al. [12] and Brosens, Pijnenborg, and Benagiano [68]. FGR, fetal growth restriction; PPH, postpartum hemorrhage; PPROM, preterm premature rupture of membranes; SGA, small for gestational age.

**Figure 9:**
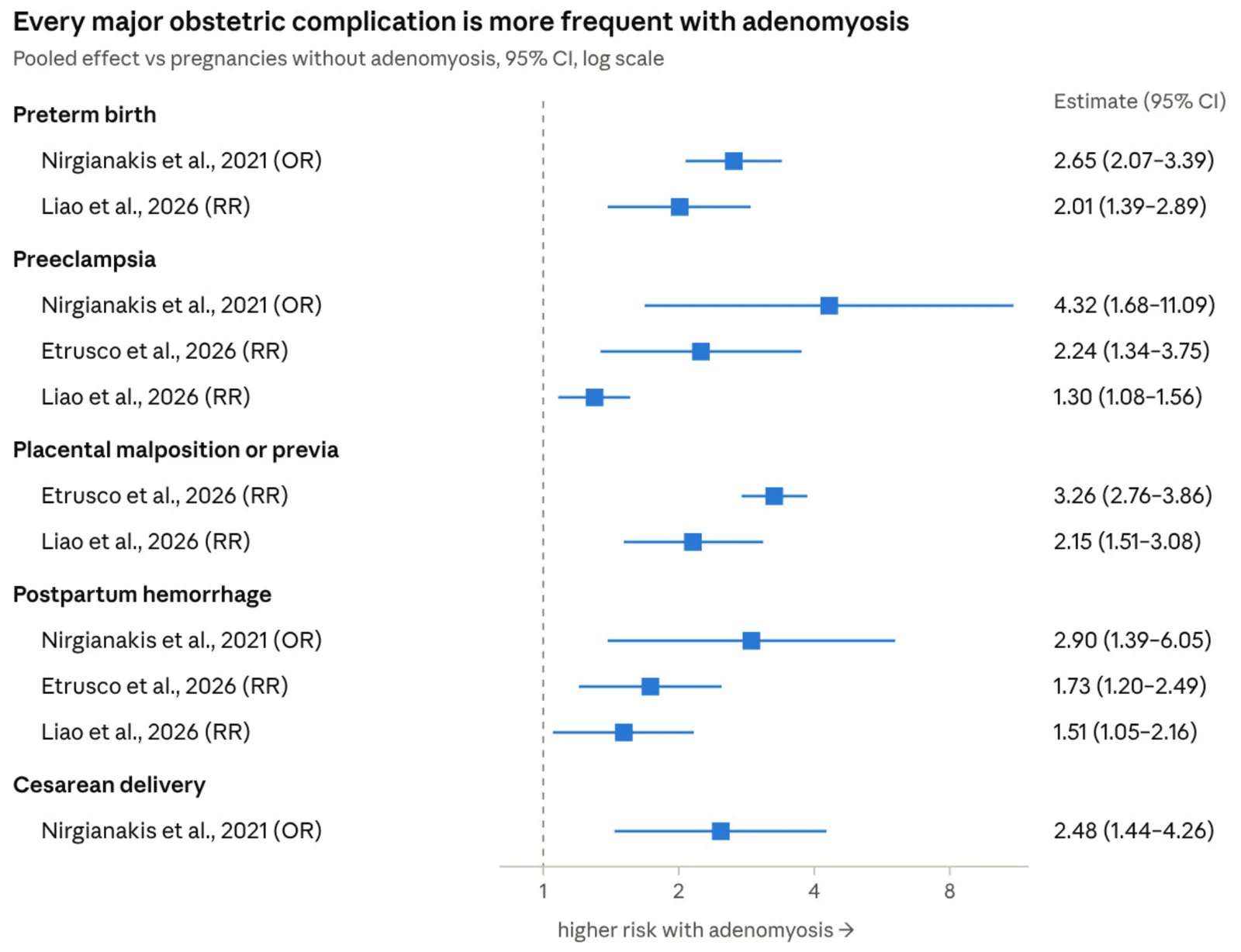
Obstetric complications in pregnancies with versus without adenomyosis across three meta-analyses. Data from Nirgianakis et al. [37] (odds ratios, OR), Etrusco et al. [78], and Liao et al. [85] (relative risks, RR), with 95% confidence intervals (CI) on a logarithmic scale. The dashed vertical line marks the null value (1), and the arrow indicates the direction of higher risk with adenomyosis.

**Figure 10:**
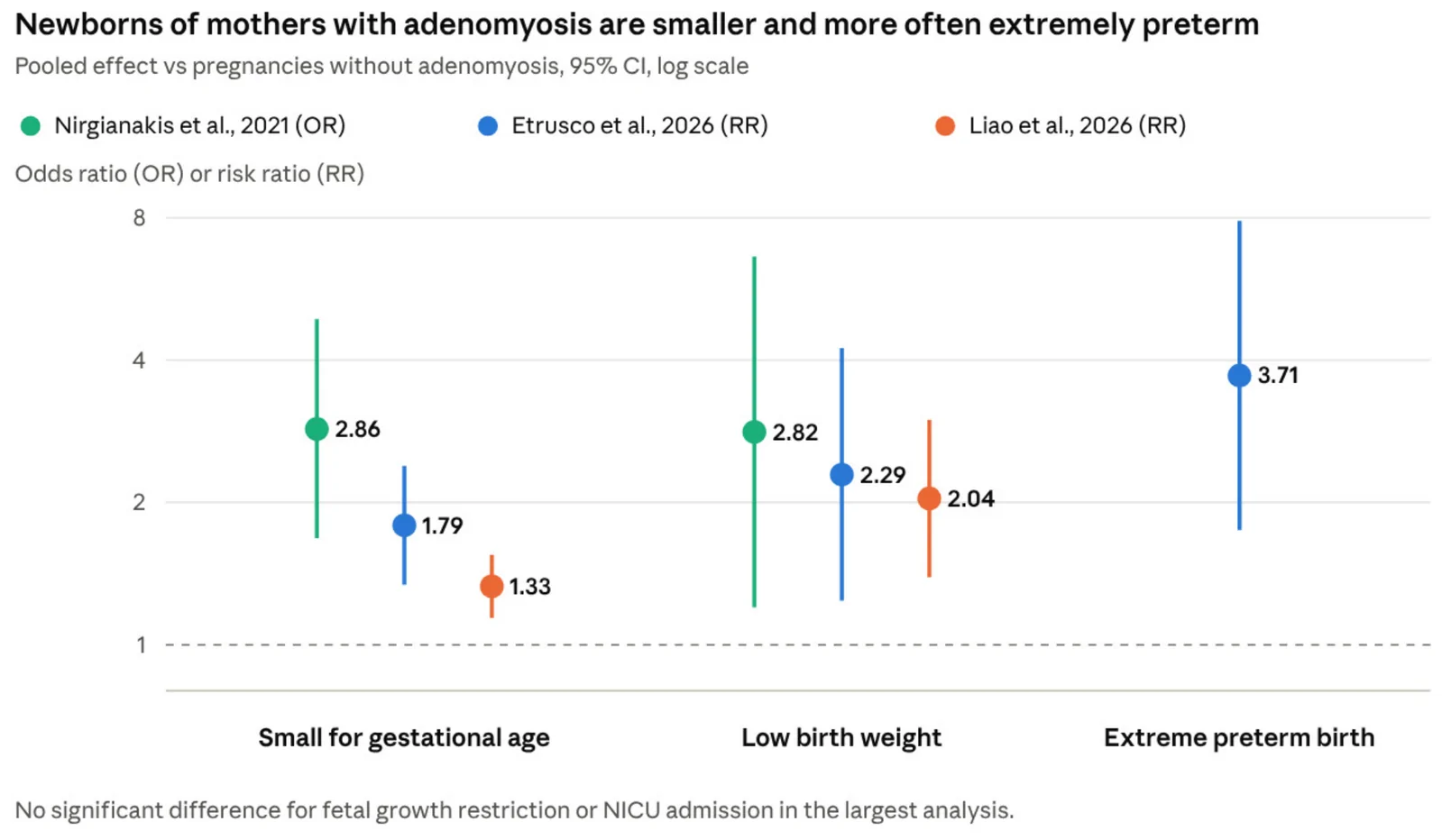
Neonatal outcomes in pregnancies with versus without adenomyosis. Data from Nirgianakis et al. [37] (odds ratios, OR), Etrusco et al. [78], and Liao et al. [85] (relative risks, RR); bars show 95% confidence intervals (CI) on a logarithmic scale; colors identify the three meta-analyses, as shown in the legend, and the dashed horizontal line marks the null value (1). No significant difference was found for fetal growth restriction or NICU admission in the largest analysis [78]. NICU, neonatal intensive care unit.

**Figure 11:**
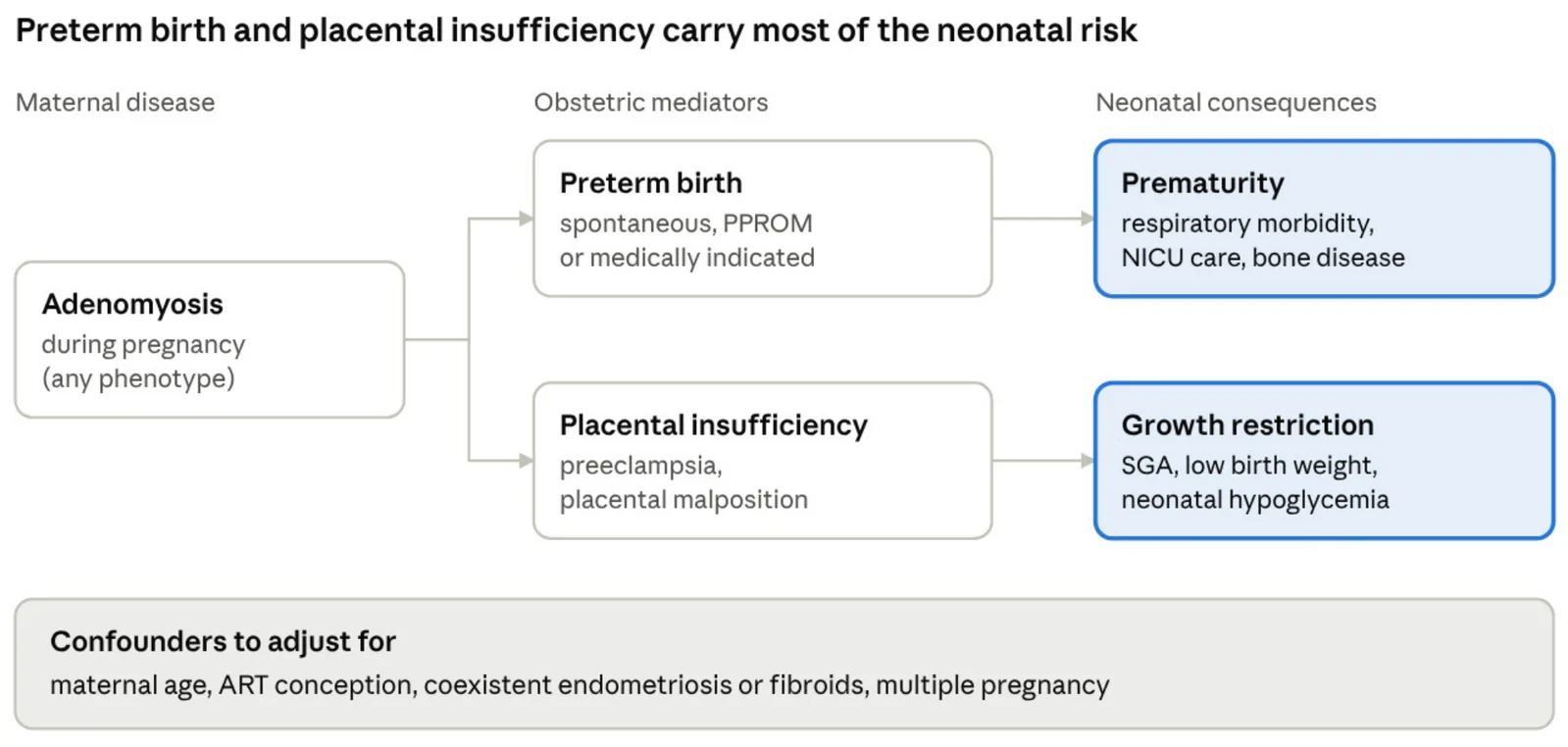
Pathways from maternal adenomyosis to neonatal outcomes and the main confounders. Original schematic synthesizing Nirgianakis et al. [37] and Etrusco et al. [78]. Arrows indicate the proposed causal direction, from maternal disease through obstetric mediators to neonatal consequences (blue boxes); the gray box lists the main confounders. ART, assisted reproductive technology; NICU, neonatal intensive care unit; PPROM, preterm premature rupture of membranes; SGA, small for gestational age.

**Figure 12:**
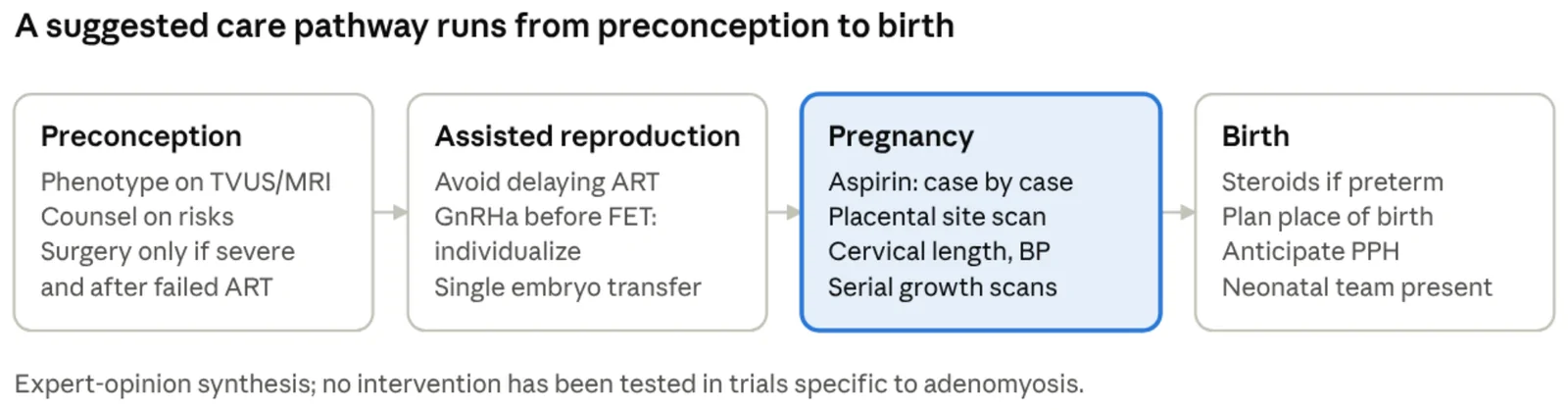
Suggested clinical pathway for women with adenomyosis who wish to conceive. Original schematic by the author based on the evidence reviewed; aspirin is an individualized option, not routine care (Section 4.5). Arrows indicate the chronological sequence of the four stages, and the blue box highlights pregnancy, the stage in which surveillance is concentrated. ART, assisted reproductive technology; BP, blood pressure; FET, frozen embryo transfer; GnRHa, gonadotropin-releasing hormone agonist; MRI, magnetic resonance imaging; PPH, postpartum hemorrhage; TVUS, transvaginal ultrasound.

**Table 1:** Classification systems for adenomyosis.

System	Method	Categories	Reference
Bird et al. (1972)	Histology	Depth: grade I sub-basal, grade II up to mid-myometrium, grade III beyond mid-myometrium; degree: slight (1–3 foci per low-power field), moderate (4–9), marked (≥10)	[13]
Kishi et al. (2012)	MRI	Type I intrinsic; type II extrinsic; type III intramural; type IV indeterminate	[14]
Chapron et al. (2017)	MRI	Diffuse adenomyosis (junctional zone [JZ] ≥12 mm and JZ/myometrium ratio > 40%); focal adenomyosis of the outer myometrium (FAOM)	[8]
Grimbizis’s system as reported by Gordts et al. (2018)	Clinical and histological	Diffuse; focal (adenomyoma, cystic adenomyosis); polypoid adenomyoma (typical, atypical); special forms	[15]
Bazot and Daraï (2018)	TVUS and MRI	Internal adenomyosis; external adenomyosis; adenomyoma (may coexist)	[16]
Van den Bosch et al. (2019), MUSA	TVUS	Location; type (focal or diffuse); cystic or non-cystic; layer (JZ, middle myometrium, subserosa); extent (<25%, 25–50%, >50%); size	[17]
Lazzeri et al. (2018), Exacoustos et al. (2020)	TVUS	Type and degree of JZ and outer myometrium involvement, each scored 1–4; total score mild (1–3), moderate (4–6), severe (≥7)	[18,19]
Keckstein et al. (2021), #Enzian	Surgery, TVUS and MRI	FA: presence of adenomyosis recorded with endometriosis	[20]
Signorile et al. (2019)	Histology and immunohistochemistry	Microscopic adenomyosis: endometrial glands without stroma in the myometrium, the earliest form	[21]

JZ, junctional zone; MRI, magnetic resonance imaging; MUSA, Morphological Uterus Sonographic Assessment; TVUS, transvaginal ultrasound. In “#Enzian”, the symbol # is part of the official name of the classification and does not indicate a footnote.

**Table 2:** Endometrial adhesion and receptivity molecules altered in adenomyosis.

Molecule	Role in Implantation	Finding in Adenomyosis	Reference
L-selectin ligand (MECA-79)	Luminal epithelial ligand for trophoblast L-selectin; initial apposition	Downregulated in luminal epithelium in the mid-secretory phase	[49]
Integrin αvβ3 (β3 subunit)	Epithelial receptor for firm trophoblast attachment; marker of the receptive window	Reduced in the implantation window; no difference in a later study	[50,51]
Osteopontin	Ligand that bridges integrin αvβ3 and the trophoblast	Reduced in the implantation window (stromal in the later study)	[50,51]
E-cadherin	Epithelial cell–cell adhesion and polarity	Lost during estrogen-induced epithelial–mesenchymal transition (indirect evidence)	[52]
LIF and LIF receptor	Cytokine that induces receptivity through STAT3 signaling	Reduced in endometrium and uterine fluid; lower LIF receptor and STAT3 activation	[53,54]
HOXA10	Transcription factor that controls the receptivity program	Reduced in secretory-phase endometrium	[51,55]

HOXA10, homeobox A10; LIF, leukemia inhibitory factor; STAT3, signal transducer and activator of transcription 3.

**Table 4:** Labor and delivery complications in women with adenomyosis.

Complication	Main Findings	Reference
Failure to progress (labor dystocia)	aOR 1.24 (95% CI 1.12–1.37); prolonged or protracted labor contributing to postpartum hemorrhage	[34,102]
Emergency cesarean delivery	aOR 1.54 (95% CI 1.41–1.68)	[34]
Cesarean delivery (all)	20.9% versus 8.3% (emergency cesarean only); pooled OR 2.48; 40% versus 26%	[33,37,102]
Fetal malpresentation	Pooled OR 3.05	[37]
Operative vaginal delivery and oxytocin augmentation	Not increased in the largest cohort	[34]
Retained placenta	aOR 1.29 (95% CI 1.10–1.48)	[34]
Postpartum hemorrhage (uterine atony, placental bed bleeding)	aOR 1.23 (95% CI 1.10–1.38); 13% versus 1%	[34,102]
Uterine rupture	0.2% versus 0.01%; higher risk after adenomyomectomy (Section 5.3)	[33,103]

aOR, adjusted odds ratio; CI, confidence interval; OR, odds ratio.

**Table 6:** Uterine rupture in women with adenomyosis.

Setting	Timing	Main Features	Reference
Unscarred uterus, not in labor	18 weeks to term (22 weeks in the index case)	Transmural adenomyosis at the rupture site; frequent hysterectomy and fetal loss	[112]
Unscarred uterus at term	Term	Posterior wall rupture; maternal cardiac arrest	[113]
Unscarred uterus in labor	39 weeks, induced labor	Adenomyosis at the rupture site	[114]
Population data	Any	0.2% versus 0.01% in one registry; not significant in the meta-analysis, which included only this registry for the outcome	[33,78]
After adenomyomectomy	16–35 weeks, before labor	5 of 304 pregnancies (2.0% of women); 13 of 363 deliveries (3.6%) in a worldwide review; up to 12.5% per patient in small series; placenta over the scar; fetal deaths	[109,115,116]
After adenomyomectomy and ICSI	25 weeks	Fetus expelled into the abdominal cavity; infant survived	[118]

ICSI, intracytoplasmic sperm injection.

**Table 7:** Conservative surgical options for adenomyosis and reproductive outcomes.

Treatment	Main Indication	Pregnancy Outcome	Main Obstetric Risks	Reference
Complete excision (adenomyomectomy)	Focal disease External disease	Pregnancy 60.5% (review); about 50% after any adenomyomectomy and 58.3% for focal disease (meta-analysis)	Rupture rare in focal disease; placenta accreta spectrum	[140,141,162]
Incomplete (cytoreductive) excision	Diffuse disease	Pregnancy 46.9% (review); about 34%	Rupture 6.8–12.5%	[109,141,162]
Triple-flap method (laparotomy)	Diffuse or focal disease	16 of 26 conceived; 14 delivered	No rupture in the original series	[167]
Double-flap method	Diffuse disease	56 of 137 conceived (40.9%); 45 live births	No rupture in 62 pregnancies	[161]
Excision together with the deep endometriosis nodule	External adenomyosis of the posterior wall	Clinical pregnancy 36.0% (9/25)	No rupture in the series; one rupture in labor in a case report	[149,151]

**Table 8:** Summary of the evidence: established associations, guideline-supported measures, proposed mechanisms, and untested proposals.

Category and Item	Main Evidence	Status	Section
**Established associations**			
Lower clinical pregnancy and live birth, higher miscarriage after IVF	Four meta-analyses [35,36,37,38]; with donor oocytes, mainly higher miscarriage [65,66,67]; lower implantation and live birth also after euploid transfer [44,45]	Consistent; observational	Section 3.2, Section 3.7 and Section 4.2
Preeclampsia, preterm birth, PPROM, placental malposition	Meta-analyses [37,78,85]; population cohorts [34,89]	Low to very low certainty	Section 4.3 and Section 4.4
Placenta accreta spectrum and abruption	Cohorts [90,91]; far more frequent after adenomyomectomy [92]	Observational	Section 4.4
Cesarean delivery, failure to progress, retained placenta, postpartum hemorrhage	ADENO cohort [34]; registry [33]; meta-analysis [37]	Observational	Section 5.1
SGA, low birth weight, extreme preterm birth	Meta-analyses [37,78,85]	Low to very low certainty	Section 6.1
Pregnancy reported in approximately half of women after uterus-sparing surgery in selected cohorts; rupture 2–12.5% per patient (3.6% of deliveries)	Meta-analysis [140]; series [109,115,116]	Observational, heterogeneous	Section 7.1
**Measures supported by existing guidelines (not specific to adenomyosis)**			
Timing of delivery for placenta previa, placenta accreta spectrum and FGR	ACOG, RCOG, SMFM [104,105,106,107,108]	Guideline	Section 5.2
Antenatal corticosteroids at 24+0–33+6 weeks; late preterm according to ALPS criteria	ACOG, Cochrane, ALPS [120,121,122]	Guideline	Section 6.2
Low-dose aspirin for guideline-listed risk factors	ACOG, NICE [97,98]	Guideline	Section 4.5
Folic acid for all women; treatment of documented vitamin D deficiency	CDC [76]; ACOG [130]	Guideline	Section 4.1 and Section 6.3
**Proposed mechanisms (hypotheses)**			
Defective junctional zone spiral artery remodeling	Histological and observational data [12,68]	Not demonstrated in pregnancies with adenomyosis	Section 4.1
Reduced endometrial adhesion and receptivity molecules	Small tissue studies [49,50,51,52,53,54,55]	Hypothesis	Section 3.5
Abnormal myometrial contractility in labor	Indirect evidence [34,101,102]	Hypothesis; never measured in labor	Section 5.1
Hyperhomocysteinemia and low folate	One small study [69]; analogy with the general population [70,71,72,73,74,75]	Hypothesis	Section 4.1
Extra-ovarian AMH raising serum AMH	Tissue expression only [61,62,63]	Speculative	Section 3.6
Primary ovarian impairment	Animal model of endometriosis-like disease [59]	Not tested in adenomyosis	Section 3.6
**Proposals specific to adenomyosis, untested (not recommendations)**			
Low-dose aspirin for adenomyosis alone	Extrapolation from other high-risk groups [95,96,97,98,99,100]	Individualized option; trial needed	Section 4.5
Screening for vitamin D or homocysteine–folate status	Association data only [69,70,71,72,73,74,75,125,126,127,128,129]	Research question	Section 4.1 and Section 6.3
Routine GnRHa pretreatment before ART	No live birth benefit [43,46,47,48]	Individualize	Section 3.3
Surgery in preference to medical treatment or ART	Non-randomized comparisons [30,117,168,169,170]	Not established; trial needed	Section 7.1
Fixed gestational age for delivery after adenomyomectomy	Surgical series [109,110,111]	Individualize (35–38 weeks)	Section 5.2
Neonatal bone disease and birth fractures linked to adenomyosis	Inferred from prematurity [123,124,125,126,127,128,129,131,132,133]	Research question	Section 6.3

ALPS, Antenatal Late Preterm Steroids trial; AMH, anti-Müllerian hormone; ACOG, American College of Obstetricians and Gynecologists; ART, assisted reproductive technology; CDC, Centers for Disease Control and Prevention; FGR, fetal growth restriction; GnRHa, gonadotropin-releasing hormone agonist; IVF, in vitro fertilization; NICE, National Institute for Health and Care Excellence; PPROM, preterm premature rupture of membranes; RCOG, Royal College of Obstetricians and Gynaecologists; SGA, small for gestational age; SMFM, Society for Maternal-Fetal Medicine.

## Data Availability

No new data were created or analyzed in this study. Data sharing is not applicable to this article.
